# Barium senses subtle pore changes in a voltage-gated K^+^ channel associated with voltage sensor states and regulatory subunits

**DOI:** 10.1126/sciadv.aec6510

**Published:** 2026-07-10

**Authors:** Lei Huang, Nitzan Daus, Yuyin Wang, Guohui Zhang, Manar Khier, Ariel Ben-Bassat, Jingyi Shi, Lu Zhao, Borui Zhang, Lu Han, Yoni Haitin, Bernard Attali, Jianmin Cui

**Affiliations:** ^1^ Department of Biomedical Engineering, Center for the Investigation of Membrane Excitability Diseases, Washington University in St. Louis, MO 63130, USA.; ^2^ Department of Physiology and Pharmacology, Gray Faculty of Medical and Health Sciences and Sagol School of Neurosciences, Tel Aviv University, Tel Aviv 6997801, Israel.

## Abstract

In classic models of voltage-dependent channel activation, the pore only opens after the voltage sensor domains (VSDs) activate in an “all-or-none” fashion. Whether the VSD alters open pore properties remains unclear. Here, we examine the properties of the KCNQ1 channel pore in relation with the VSD and regulatory subunits that are located outside of the pore using barium ion (Ba^2+^) block as a probe. We find that the external Ba^2+^ block of KCNQ1 channels is voltage-dependent, and the voltage-dependent channel opening in Ba^2+^ mainly derives from voltage-dependent Ba^2+^ unblock. Open pore conformational changes indicated by the voltage dependence of Ba^2+^ unblock vary with the states of the VSD, the alterations of VSD-pore coupling, and the association with regulatory KCNE subunits. Contrary to the classic view, our results suggest that, instead of being independent of the voltage gating machinery, the pore of KCNQ1 is flexible and influenced by the subtle changes in the voltage sensor state and the environment outside of the pore.

## Teaser

The open KCNQ1 pore is flexible and fine-tuned by interactions with the voltage sensor and KCNE subunits as probed by Ba^2+^ block.

## INTRODUCTION

Voltage-gated K^+^, Na^+^, and Ca^2+^ (K_V_, Na_V_, and Ca_V_) channels generate electric signals, known as action potentials, for the function of neurons, cardiac myocytes, and muscle cells. These channels also control membrane potential and ion homeostasis in nearly all cell types to regulate cell functions and cell fate. The voltage-gated channels comprise four subunits (K_V_) or domains (Na_V_ and Ca_V_) and adopt a similar structure with a central pore formed by transmembrane helices S5 and S6 from all four subunits or domains, which is surrounded by four voltage sensor domains (VSDs) formed by transmembrane helices S1 to S4 of each subunit or domain (*1*–*5*). During voltage-dependent activation of these channels, membrane depolarization triggers conformational changes in the VSD, and the VSD movements subsequently promote the pore to open for passing K^+^, Na^+^, and Ca^2+^ ions through a process called VSD-pore coupling or electromechanical coupling (*6*, *7*).

Hodgkin and Huxley studied the generation of action potentials in squid giant axon by K^+^ and Na^+^ conductance long before ion channels were identified (*8*–*12*). In their classic models of voltage-dependent activation of the K^+^ and Na^+^ conductance, the probability of conduction is determined by voltage-dependent gates, with four identical and independent activation gates for K^+^ conductance, and three activation and one inactivation gates for Na^+^ conductance (*9*). The Hodgkin and Huxley models were adopted seamlessly to describe voltage-dependent activation of K_V_, Na_V_, and Ca_V_ channels after the molecular identification of these channels, with the gates corresponding to VSDs and the conductance corresponding to pore opening (*13*–*19*).

In the classic models of voltage-dependent channel activation, the pore is triggered open by VSD activation in an “all-or-none” fashion (*9*–*12*, *20*, *21*). The open pore features a constant conductance and is independent of the VSD states once it is open. Thus, the ionic current through the pore varies with voltage only by the changes in the electrochemical driving force but not by any conformational change of the pore. In some K^+^ channels, discrete substate conductance levels were observed (*22*–*25*), but whether these substates of the open pore are regulated by VSD conformations is not clear.

The KCNQ1 channel (or K_V_7.1) belongs to the family of K_V_ channels that is expressed in many organs such as the heart, brain, and various epithelial tissues and serves important physiological functions, including the regulation of cardiac action potential duration and heart rate, hearing, and epithelial salt homeostasis (*26*). In the heart, coassembly of the KCNQ1 α subunit with the KCNE1 regulatory subunit forms the slow voltage-dependent I_Ks_ potassium current that repolarizes the cardiac action potential (*27*–*30*). On the other hand, association of KCNQ1 with the KCNE3 regulatory subunit in epithelial tissues forms a constitutively open channel at physiological voltages to transport K^+^ (*31*). Reflecting its physiological importance, mutations in the KCNQ1 protein are associated with the cardiac long QT syndrome, atrial fibrillation, and deafness (*32*–*34*).

Previously, we reported that KCNQ1 features a two-open-state gating mechanism, showing that its VSD can trigger pore opening when activated to both intermediate (I) and fully activated (A) states, leading to two conducting open states: the fast intermediate open (IO) and the slow fully activated open (AO) (*35*–*41*). The IO and AO states exhibited different ion permeability, indicating different conformations of the pore in these two open states (*37*). We demonstrated that the stepwise VSD activation of KCNQ1 induces pore opening at the I and A states via distinctive sets of coupling interactions (*39*, *40*). This mechanism is, in general, consistent with the canonical electromechanical coupling such that during gating, the voltage-driven movement of the S4 helix leads to a translation of the intracellular linker S4-S5, which interacts with the pore to couple the VSD motion to pore opening (*6*, *7*, *42*). However, in KCNQ1 channels, we found that the interactions of the C terminus of the S4-S5 linker with S6 in the same subunit, as previously found in Shaker K^+^ channels, are important for the VSD-pore coupling when the VSD is at both the I and A state; additional interactions of the N terminus of the S4-S5 linker with S5 and S6 of a neighboring subunit are required for the opening of the pore when the VSD is at the A state. This mechanism is summarized as a “hand-and-elbow” model such that both the hand (C terminus of the S4-S5 linker) and elbow (N terminus of the S4-S5 linker) grab and nudge the pore to open the channel (*40*). These results indicate that in voltage-dependent activation of KCNQ1, the pore may not remain the same and open in the “all-or-none” fashion. Rather, the pore responds to the discrete I and A states of the VSD and opens to different IO and AO conformations via different sets of VSD-pore interactions. In addition, the KCNQ1 channel pore may partially open by the activation of individual subunits, further deviating from the “all-or-none” opening (*43*, *44*).

We previously showed that extracellular Ba^2+^ exerts on KCNQ1 channel a series of complex effects, including a pore block as well as alterations of voltage-dependent opening of the channel (*45*, *46*). In the present work, we used Chinese hamster ovary (CHO) cells and *Xenopus* oocytes heterologous expression systems to further study the effects of external Ba^2+^ on KCNQ1 channel permeation and gating properties. Our results show that Ba^2+^ does not affect the VSD activation, although the KCNQ1 current in Ba^2+^ exhibited altered voltage dependence. Rather, Ba^2+^ block of KCNQ1 is voltage dependent, with a weaker inhibition at positive potentials. Because of its voltage-dependent block, Ba^2+^ produces a substantial right-shift in the voltage dependence of channel opening, slows down the rising, and accelerates the falling kinetics of the currents. Using Ba^2+^ block as a probe, we find that the voltage dependence of Ba^2+^ block varies with the states of the VSD, the alterations of VSD-pore coupling, and the association with regulatory KCNE subunits, suggesting that the open pore conformation changes under these settings. Each condition produces a unique Ba^2+^ sensitivity rather than a discrete pattern, underscoring that the open KCNQ1 pore is highly flexible and finely tuned by its interactions with the voltage sensor and auxiliary subunits.

## RESULTS

### Barium produces a voltage-dependent pore block and alters channel opening in KCNQ1

**Fig. 1.**
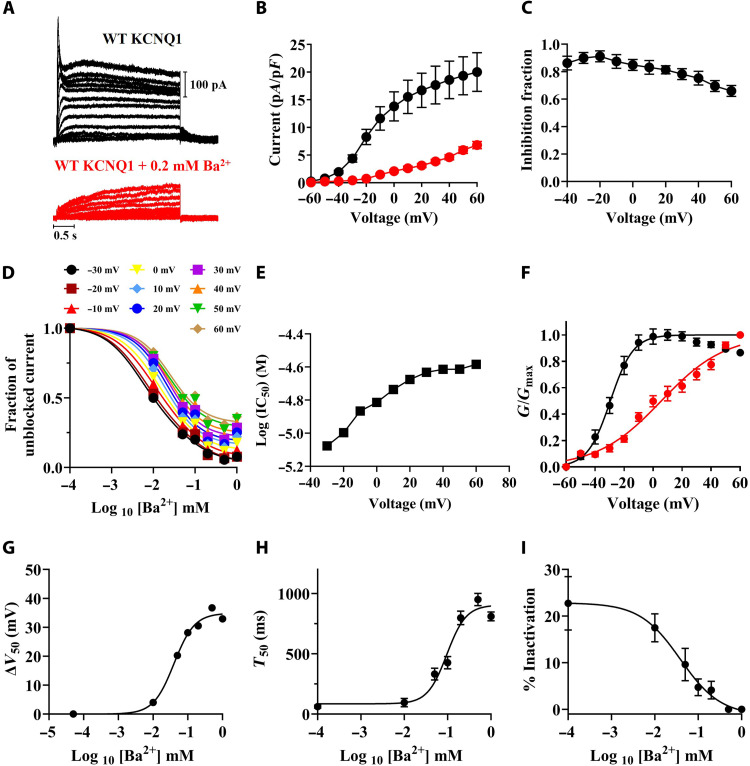
External Ba^2+^ caused a concentration- and voltage-dependent block of WT KCNQ1 in CHO cells. (**A**) Representative WT KCNQ1 current traces in the absence (black) and presence of 0.2 mM Ba^2+^ (red). (**B**) *I*-*V* relationships of WT KCNQ1 before and after 0.2 mM Ba^2+^ (*n* = 9). (**C**) Inhibition fraction-voltage relationship of WT KCNQ1 (*n* = 9). (**D**) Concentration- and voltage-dependent block of WT KCNQ1. The curves ranging from −30 to 0 mV were best fitted using an equation that assumes the presence of two binding sites. The corresponding IC_50_ values were as follows: 4.5 and 55.6 μM at −30 mV, 5.0 and 63.3 μM at −20 mV, 6.6 and 50.0 μM at −10 mV, and 9.2 and 34.4 μM at 0 mV (*n* = 7 to 9). The curves ranging from +10 to +60 mV were best fitted with an equation that assumes a single binding site. The corresponding IC_50_ values were as follows: 18.4 μM at +10 mV, 21.1 μM at +20 mV, 23.3 μM at +30 mV, 24.3 μM at +40 mV, 24.4 μM at +50 mV, and 26.1 μM at +60 mV (*n* = 7 to 9). (**E**) Relation of barium affinity expressed as log (IC_50_) (M) versus voltage. (**F**) *G*-*V* relationships of WT KCNQ1 before and after 0.2 mM Ba^2+^. *V*_1/2_ and slope factor (mV): −29.5 ± 1.8 and 7.9 ± 0.6 for WT KCNQ1; 5.6 ± 3.7 and 22.1 ± 1.4 for WT KCNQ1 with 0.2 mM Ba^2+^ (*n* = 9; *P* < 0.0001 for both *V*_1/2_ and slope factor). (**G** to **I**) Concentration dependence of Ba^2+^ effect on Δ*V*_1/2_ (G), channel opening kinetics (*T*_50_) (H), and channel inactivation (I) (*n* = 7 to 9).

First, we characterized the external Ba^2+^ pore block on KCNQ1 in transfected CHO cells. The steady-state pore block (Fig. 1, A and B) is reached within 3 min of external Ba^2+^ application (0.2 mM BaCl_2_) using our perfusion system. Steady-state Ba^2+^ block exhibited a strong voltage dependence. The inhibition fraction measured as a function of voltage at 0.2 mM Ba^2+^ showed less block at more depolarized membrane potentials (Fig. 1C). Increasing concentrations of BaCl_2_ progressively block the channels, but even at saturating concentrations, Ba^2+^ did not completely block the currents, leaving an unblocked component (Fig. 1D). The concentration of Ba^2+^ at half-blocked currents [median inhibitory concentration (IC_50_)] as well as the unblocked component increased with increasing membrane potential (Fig. 1, C to E), consistent with Ba^2+^ block being voltage dependent.

We observed that Ba^2+^ also altered voltage-dependent opening of KCNQ1 channels. The steady-state conductance-voltage (*G*-*V*) relationship exhibited a right-shift in the presence of Ba^2+^, which was also accompanied by a shallower steepness (Fig. 1F). The *G*-*V* shift became larger with higher Ba^2+^ concentrations, yielding a median effective concentration (EC_50_) of 39.9 μM (Fig. 1G). External Ba^2+^ also slowed down the opening kinetics of channels (Fig. 1A). The slowing of channel opening depended on Ba^2+^ concentration and yielded an EC_50_ of 95.8 μM (Fig. 1, A and H). In addition, external Ba^2+^ also inhibited KCNQ1 inactivation (Fig. 1, A and I). The concentration-dependent curve for Ba^2+^ inhibition of inactivation yields an IC_50_ of 38.6 μM (Fig. 1I).

### Barium alters pore opening by voltage-dependent block

**Fig. 2.**
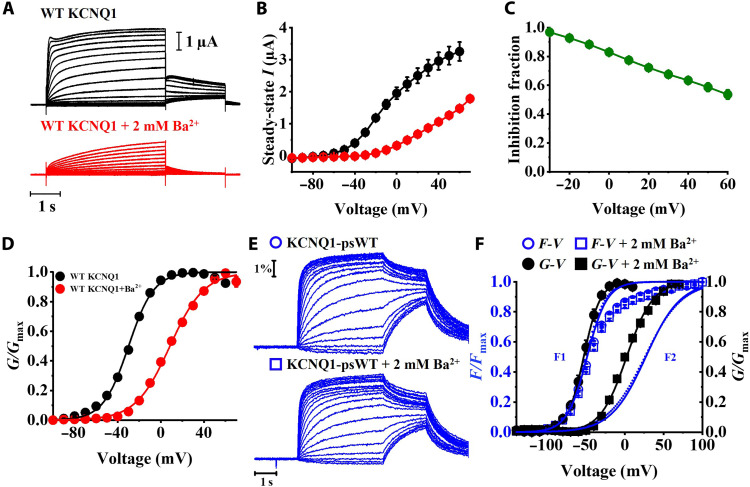
External Ba^2+^ produced a voltage-dependent block of WT KCNQ1 without affecting its VSD movement in *Xenopus* oocytes. (**A**) Representative WT KCNQ1 current traces in the absence (black) and presence of 2 mM Ba^2+^ (red) from the same oocyte. (**B**) *I*-*V* relationships of WT KCNQ1 before and after 2 mM Ba^2+^ (*n* = 6). (**C**) Inhibition fraction of WT KCNQ1 (*n* = 6). (**D**) *G*-*V* relationships of WT KCNQ1 before and after 2 mM Ba^2+^. *V*_1/2_ and slope factor (mV): −29.683 ± 0.723 and 11.747 ± 0.465 for WT KCNQ1; 8.802 ± 1.349 and 16.354 ± 0.500 for WT KCNQ1 with 2 mM Ba^2+^ (*n* = 6; *P* < 0.0001 for both *V*_1/2_ and slope factor). (**E**) Representative fluorescence signals from KCNQ1-psWT before (blue open circle) and after (blue open square) 2 mM Ba^2+^ from the same oocyte. (**F**) *F*-*V* and *G*-*V* relationships. *F*-*V* relationships were fitted by double Boltzmann equation with *F1* and *F2*. *F1* component with *V*_1/2_ and slope factor (mV): −50.971 ± 0.763 and 12.889 ± 0.216 for KCNQ1-psWT (blue dotted line); −48.821 ± 0.979 and 13.033 ± 0.707 for KCNQ1-psWT with 2 mM Ba^2+^ (blue line). *F2* component with *V*_1/2_ and slope factor (mV): 25.683 ± 3.008 and 25.997 ± 3.487 for KCNQ1-psWT (blue dotted line); 26.967 ± 6.957 and 25.075 ± 4.832 for KCNQ1-psWT with 2 mM Ba^2+^ (blue line). *n* = 4; *P* > 0.05 for *V*_1/2_ and slope factor for *F1* and *F2*. *G*-*V* relationships before (black solid circle) and after 2 mM Ba^2+^ (black solid square). *V*_1/2_ and slope factor (mV): −52.759 ± 2.115 and 10.462 ± 0.203 for KCNQ1-psWT; 2.622 ± 1.379 and 17.194 ± 0.586 for KCNQ1-psWT with 2 mM Ba^2+^. *n* = 4; *P* = 0.0002 for *V*_1/2_ and *P* = 0.0007 for slope factor.

Did our observed changes in voltage-dependent opening of KCNQ1 channels produced by external Ba^2+^ exposure derive from any alteration of voltage-dependent gating induced by Ba^2+^? To explore this possibility, we measured whether external Ba^2+^ could affect the VSD motion using the voltage-clamp fluorometry (VCF) techniques to study KCNQ1 channels expressed in *Xenopus* oocytes. Similar to the effects observed in transfected CHO cells, external Ba^2+^ (2 mM) application produced a voltage-dependent pore block with increasing unblock at depolarized potentials (Fig. 2, A to C). Ba^2+^ also slowed down the opening kinetics, accelerated channel closing at repolarization, and induced a right-shift in the voltage dependence of channel opening accompanied by a shallower slope (Fig. 2, A and D). Using VCF, we measured VSD movements and pore opening simultaneously with and without Ba^2+^ application. As in previous studies, a fluorophore (Alexa Fluor 488) specifically labeled Gly^219^→Cys (G219C) in the S3-S4 linker of KCNQ1–pseudo-wild type (KCNQ1-psWT) (C214A/G219C/C331A) sensed the local environment changes in response to membrane voltages, and the fluorescence changes reflected two-step VSD movements (Fig. 2E), while the ionic currents reported pore opening (fig. S1A) (*43*, *47*, *48*). The fluorescence-voltage (*F*-*V*) relationship could be well fitted by a double Boltzmann distribution with two components, *F1*-*V* and *F2*-*V* (Fig. 2F), which report the voltage dependence of VSD activation from resting (R) state to the intermediate (I) state and from the intermediate state to the fully activated (A) state, respectively. *F1*-*V* and *F2*-*V* relationships spanned large voltage ranges, with *F2* starting at around −50 mV, indicating that the VSD moved to the activated state at voltages more positive than −50 mV (*35*–*37*). The pore of KCNQ1 channel was open at both intermediate and activated VSD states, as indicated by the *G*-*V* relationship (Fig. 2F), generating the IO and AO states. Ba^2+^ also produced a voltage-dependent pore block (fig. S1) and shifted the *G*-*V* relationship to the right (Fig. 2F) on KCNQ1-psWT. External Ba^2+^ did not affect the voltage dependence and the relative proportions of the *F1* or *F2* components (Fig. 2, E and F), suggesting that Ba^2+^ did not affect VSD movements.

**Fig. 3.**
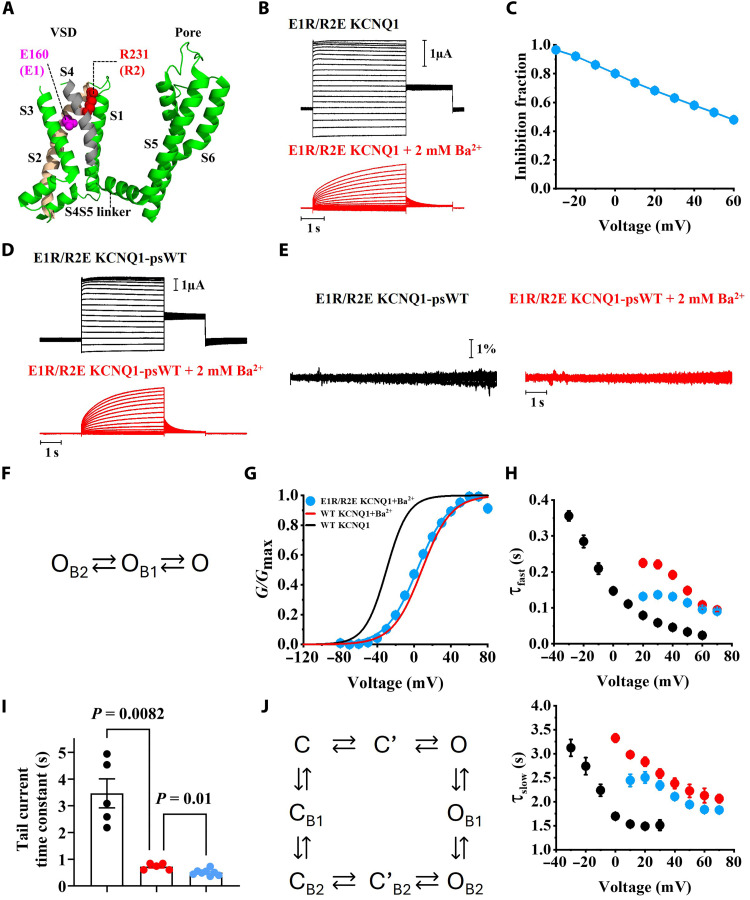
Voltage-dependent channel opening in external Ba^2+^ derived from voltage-dependent Ba^2+^ unblock. (**A**) Residues, E160 (E1) and R231 (R2), marked in one subunit of KCNQ1 channel from human KCNQ1 cryo–electron microscopy structure (Protein Data Bank: 6UZZ). Residue E1 is highlighted in magenta and located in the S2 segment (wheat), while residue R2 is labeled in red on the S4 segment (gray). Other segments and linkers are noted in green, and the structure below the S6 segment has been omitted. (**B**) Representative E1R/R2E KCNQ1 current traces with and without 2 mM Ba^2+^ from the same *Xenopus* oocyte. (**C**) Inhibition fraction of E1R/R2E KCNQ1 (*n* = 8). (**D** and **E**) Representative current traces (D) and fluorescence signals (E) recorded simultaneously from E1R/R2E KCNQ1-psWT before and after 2 mM Ba^2+^ from the same oocyte. (**F**) Two blocked states and voltage-dependent transitions in E1R/R2E KCNQ1. (**G**) *G*-*V* relationship of E1R/R2E KCNQ1 with 2 mM Ba^2+^ from (B) was fitted by single Boltzmann equation with *V*_1/2_ and slope factor (mV) of 3.426 ± 1.535 and 16.570 ± 0.334 (*n* = 8). The *G*-*V* relationships of WT KCNQ1 before and after 2 mM Ba^2+^ from Fig. 2D were reproduced for comparison. The color code is the same in (G) to (I). (**H**) Voltage dependence of open time constants τ-fast and τ-slow for E1R/R2E KCNQ1 with 2 mM Ba^2+^ (Fig. 3B) and for WT KCNQ1 before and after 2 mM Ba^2+^ (Fig. 2A). (**I**) Tail current time constant (τ) for E1R/R2E KCNQ1 channel in the presence of 2 mM Ba^2+^ is 0.503 ± 0.040 (*n* = 8), and for WT KCNQ1 channel is 3.466 ± 0.543 (*n* = 5) without 2 mM Ba^2+^ and 0.726 ± 0.054 (*n* = 5) with 2 mM Ba^2+^. (**J**) Minimum mode for voltage-dependent opening of WT KCNQ1 with external Ba^2+^.

To further examine whether Ba^2+^ altered voltage-dependent gating, which contributed to our observed changes in voltage-dependent opening of KCNQ1, we studied external Ba^2+^ (2 mM) effects on a mutant KCNQ1, E1R/R2E. E1 and R2 are residues E160R and R231E in S2 and S4 of KCNQ1 (Fig. 3A and fig. S2), respectively, and the double mutation makes the channel constitutively open by arresting the VSD in the intermediate (I) state; thus, the channel opens in the IO state (*35*, *37*). We found that upon external Ba^2+^ application, the channels were blocked, and the opening became time and voltage dependent (Fig. 3B and fig. S3A). Ba^2+^ also induced a tail current (Fig. 3B). The fractional blocking was voltage dependent, with fewer channels blocked at more depolarized voltages (Fig. 3C). VCF recordings did not detect any fluorescence change during voltage steps either with or without external Ba^2+^ (Fig. 3, D and E, and fig. S4) (*41*), supporting the finding that the VSD was arrested in the I state and did not move during measurements. Thus, the robust voltage dependence of channel opening and closing could not derive from modulation of VSD activation nor VSD-pore coupling, but only from voltage-dependent Ba^2+^ unblock and reblock as described in Fig. 3F.

In Fig. 3F, O is the open state, O_B1_ and O_B2_ are Ba^2+^ blocked states, and the transitions among these states are voltage dependent. Channel opening increased during depolarization due to slow Ba^2+^ unblock and decreased when the voltage returned to the more negative potential due to fast Ba^2+^ reblock of the channel. The voltage-dependent Ba^2+^ block was measured as the steady-state *G*-*V* relationship (Fig. 3G). The time course of unblock could be fitted with a two-exponential function (Fig. 3H), suggesting that there were at least two blocked states. The time course of reblock was fitted with a single exponential function (Fig. 3I). These properties of Ba^2+^ block may derive from the voltage dependence of Ba^2+^ binding at different sites and interactions with permeant K^+^ ions (*49*–*53*).

For WT KCNQ1 channels, Ba^2+^ shifted *G*-*V* relationship, slowed down opening kinetics, and accelerated closing kinetics in a similar manner with Ba^2+^ block of mutant E1R/R2E (Figs. 2A and 3, B and G to I). Because KCNQ1 channels open primarily to the IO state, these results can be interpreted approximately by Fig. 3J, where the voltage-dependent activation of the channel involves two closed states, C and C′, and an open state, O, with voltage-dependent transitions, since the activation kinetics of KCNQ1 could be fitted with a two-exponential function (*37*, *54*, *55*); and Ba^2+^ can block both closed (C_B1_, C_B2,_ and C′_B2_) and open (O_B1_ and O_B2_) channels.

Figure 3J is the minimum model that can illustrate KCNQ1 channel voltage-dependent opening in the presence of external Ba^2+^. We omitted the closed state C′_B1_ and its transitions with C′, C′_B2_, C_B1_, and O_B1_ for the sake of model simplicity. When the membrane potential is stepped from a negative holding voltage to a positive test voltage, the channels are activated by voltage-dependent gating and Ba^2+^ unblocking due to the voltage dependence of Ba^2+^ block. These two processes promote channel opening, but the slow-unblocking of the channel seems to be prominent judging by the slow opening kinetics and the right-shift of steady-state open probability (Figs. 1 to 3). When the membrane potential is stepped back to the negative voltage, the channels deactivate via voltage-dependent gating and reblocking due to the voltage-dependent Ba^2+^ block. Reblock of the channel also appears to be dominant as reflected by the accelerated closing rate (Figs. 2A and 3, B and I). These results suggest that in the presence of external Ba^2+^, the voltage-dependent KCNQ1 opening and closing are primarily derived from the voltage dependence of Ba^2+^ block.

### The voltage dependence of Ba^2+^ block varies with VSD states and KCNE subunits

**Fig. 4.**
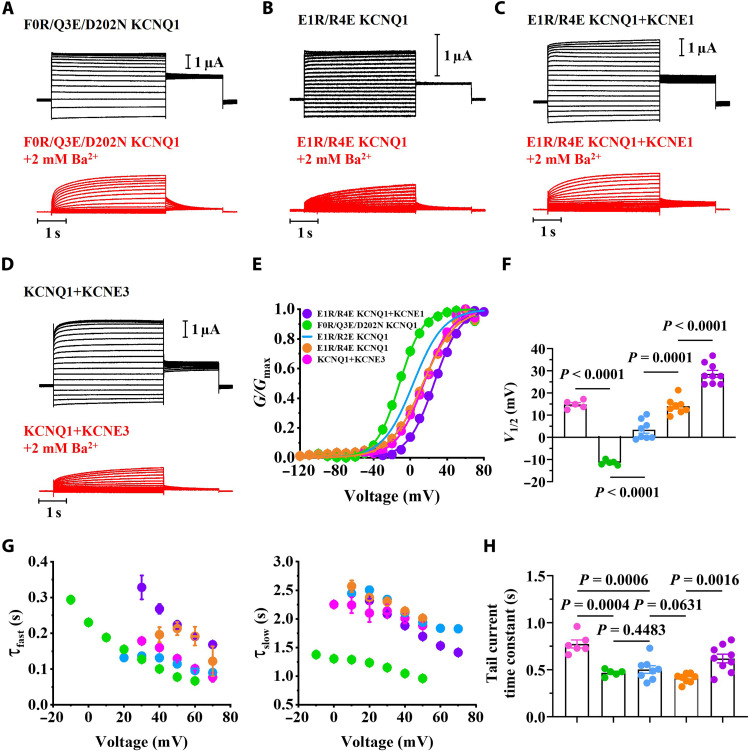
The voltage dependence of Ba^2+^ block differed depending on VSD states and KCNE subunits. (**A** to **D**) Representative current traces for constitutively open mutant channels, F0R/Q3E/D202N KCNQ1 (A), E1R/R4E KCNQ1 (B), E1R/R4E KCNQ1 + KCNE1 (C), and KCNQ1 + KCNE3 (D) expressed in *Xenopus* oocytes with recordings made in the absence (black) and presence of 2 mM Ba^2+^ (red). For each channel, the exemplar current traces before and after Ba^2+^ originated from the same oocyte. (**E**) *G*-*V* relationships of constitutively open mutant channels after 2 mM Ba^2+^. *V*_1/2_ and slope factor (mV): −11.415 ± 0.485 and 12.752 ± 0.521 for F0R/Q3E/D202N KCNQ1 (*n* = 5), 14.059 ± 1.263 and 19.196 ± 1.241 for E1R/R4E KCNQ1 (*n* = 8), 28.617 ± 1.553 and 15.193 ± 0.641 for E1R/R4E KCNQ1 + KCNE1 (*n* = 9), and 14.833 ± 0.828 and 15.803 ± 0.502 for KCNQ1 + KCNE3 (*n* = 5). The *G*-*V* relationship of E1R/R2E KCNQ1 after 2 mM Ba^2+^ from Fig. 3G was reproduced for comparison. The color code is the same for (E) to (H). (**F**) *V*_1/2_ among constitutively open mutant channels after 2 mM Ba^2+^. (**G**) Voltage dependence of activation time constants τ-fast and τ-slow for constitutively open mutant channels in the presence of 2 mM Ba^2+^. τ-fast and τ-slow of E1R/R2E KCNQ1 with 2 mM Ba^2+^ from Fig. 3H were reproduced for comparison. (**H**) Tail current time constants τ for constitutively open mutant channels in the presence of 2 mM Ba^2+^. τ (s): 0.468 ± 0.016 (*n* = 5) for F0R/Q3E/D202N KCNQ1, 0.410 ± 0.018 (*n* = 8) for E1R/R4E KCNQ1, 0.619 ± 0.046 (*n* = 9) for E1R/R4E KCNQ1 + KCNE1, and 0.774 ± 0.042 (*n* = 6) for KCNQ1 + KCNE3. The tail time constant of E1R/R2E KCNQ1 after 2 mM Ba^2+^ from Fig. 3I was reproduced for comparison.

We then examine the voltage dependence of Ba^2+^ block on other constitutively open channels (Fig. 4, A to D, and fig. S2). Our previous studies showed that another mutant F0R/Q3E/D202N also made the KCNQ1 channel open constitutively at the IO state by locking the VSD in the intermediate (I) state (Fig. 4A) (*41*). In KCNQ1, F0 is F167 in the S2 transmembrane segment, which is more toward the intracellular side than (below) E1 (E160) and forms the charge transfer center of the VSD (*56*, *57*), Q3 is Q234 in S4 below R2 (R231), and D202N is in S3 close to the charge transfer center (fig. S2). We studied Ba^2+^ block of this mutant channel and found that the channel opening became time and voltage dependent, also with the appearance of tail currents (Fig. 4A). The voltage-dependent block by Ba^2+^ to constitutively open KCNQ1 channel mutants was specific, as Ca^2+^, another divalent metal cation, did not affect the current of F0R/Q3E/D202N KCNQ1 (Fig. 4A and figs. S3B and S5). We measured the *G*-*V* relationship (Fig. 4, E and F) and opening (Fig. 4G) and closing (Fig. 4H) kinetics of F0R/Q3E/D202N KCNQ1 and found that these properties differed from that of E1R/R2E KCNQ1. Although both mutants, E1R/R2E and F0R/Q3E/D202N, arrested the KCNQ1 channels in the IO state, Ba^2+^ blocked the channel pore with different properties. These results suggest that the pore conformation in these two mutant channels may differ to result in different bindings of Ba^2+^ to the pore or interactions of Ba^2+^ with permeant K^+^ in the pore.

To further explore this finding, we studied Ba^2+^ block on other mutant KCNQ1 channels that are constitutively open and the channels in association with KCNE subunits. These channels include E1R/R4E (E160R/R237E) KCNQ1 that arrests VSD in the fully activated (A) state and leaves the channel constitutively open in the AO state (Fig. 4B and fig. S2), E1R/R4E KCNQ1 + KCNE1 (Fig. 4C and fig. S2) (*35*, *37*), and KCNQ1 + KCNE3 that constitutively opens at physiological voltages (Fig. 4D and fig. S2) (*31*, *41*). The opening of all these channels became voltage and time dependent, also with the appearance of tail currents when subjected to external Ba^2+^ blockage (Fig. 4, B to D, and fig. S3, C to E). We measured the *G*-*V* relationship and the opening and closing kinetics of these channels and found that these properties of Ba^2+^ block differ among all the constitutively open channels (Fig. 4, E to H).

**Fig. 5.**
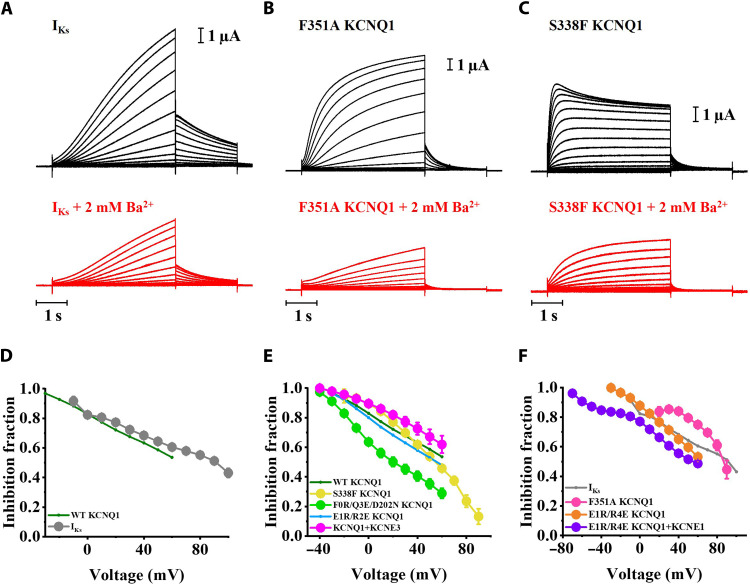
Unique voltage-dependent Ba^2+^ block depending on mutation or KCNE subunit association. (**A** to **C**) Representative current traces for I_Ks_ (KCNQ1 + KCNE1) (A), F351A KCNQ1 (B), and S338F KCNQ1 (C) expressed in *Xenopus* oocytes with recordings made in the absence (black) and presence of 2 mM Ba^2+^ (red). For each channel, the exemplar current traces before and after Ba^2+^ were from the same oocyte. (**D**) Inhibition fraction of I_Ks_ (*n* = 6). Inhibition fraction of WT KCNQ1 was reproduced from Fig. 2C. (**E**) Inhibition fraction of channels primarily opened to IO, including WT KCNQ1 (reproduced from Fig. 2C), S338F KCNQ1 (*n* = 7), F0R/Q3E/D202N KCNQ1 (*n* = 5), E1R/R2E KCNQ1 (reproduced from Fig. 3C), and KCNQ1 + KCNE3 (*n* = 5). (**F**) Inhibition fraction of channels mainly opened to AO, including I_Ks_ (reproduced from Fig. 5D), F351A KCNQ1 (*n* = 4), E1R/R4E KCNQ1 (*n* = 8), and E1R/R4E KCNQ1 + KCNE1 (*n* = 6).

We then examined the voltage-dependent Ba^2+^ block in I_Ks_ (KCNQ1 + KCNE1), which mainly opens in the AO state (Fig. 5A and fig. S2) (*37*, *41*), and additional KCNQ1 mutant channels, F351A KCNQ1 and S338F KCNQ1. In KCNQ1, F351A (Fig. 5B and fig. S2) disrupts the VSD-pore coupling when the VSD is in the intermediate (I) state and makes the channel open only at the AO, while S338F (Fig. 5C and fig. S2) disrupts the VSD-pore coupling when the VSD is in the fully activated (A) state and makes the channel open only at the IO (*37*, *39*, *40*, *54*, *58*, *59*). Exposure to external Ba^2+^ changed the steady state and kinetics of opening of these channels as compared to control recordings (Fig. 5, A to C, and fig. S6). However, because the opening of these channels was also regulated by voltage-dependent gating similar to WT KCNQ1 (Figs. 2, A to D, and 3, G to J), we did not analyze the steady state and kinetics of opening in detail to study Ba^2+^ block. Instead, we compared the voltage dependence of inhibition fraction by Ba^2+^ between KCNQ1 and I_Ks_ (Fig. 5D) and among the channels that open primarily to IO (Fig. 5E) and to AO (Fig. 5F), respectively. The inhibition fraction was a measure of Ba^2+^ blocking without the influence of voltage-dependent gating since it was the ratio of current amplitude measured at the same voltage and time point with and without external Ba^2+^. We found that the decrease in channel inhibition fraction as a function of voltage also differed among all these channels (Fig. 5, E and F). All these changes in inhibition fraction (Fig. 5, E and F) as well as the changes in *G*-*V* relationships and the kinetics of opening and closing (Fig. 4, E to H) among all the channels did not follow any recognizable pattern such as an IO or AO specific state and seemed to be unique to the mutation or KCNE subunit association.

Similar results were obtained in transfected CHO cells, where exposure to 0.2 mM Ba^2+^ led to significant voltage-dependent block, right-shift in the voltage dependence of activation, and inhibition of inactivation in S338F KCNQ1 channels (fig. S7, A to F). Does Ba^2+^ inhibit inactivation of KCNQ1 channels or just mask it? To address this question, we opened the inactivating mutant S338F by stepping the membrane potential to +60 mV and then repolarized the membrane voltage to −110 mV, a tail potential at which inactivation of KCNQ1 or human ether-à-go-go related gene (hERG) potassium channels should be recovered (fig. S7G) (*60*, *61*). If Ba^2+^ masks inactivation at depolarized potential, one should expect a larger tail current at −110 mV in the presence of Ba^2+^ than in its absence, due to putative recovery from inactivation. Measuring the current ratio with and without Ba^2+^ at +60 mV (0.35) and at −110 mV tail potential (0.44) indicates that Ba^2+^ blocks the pore and inhibits inactivation and does not mask it (fig. S7, G and H). While significant, the extent of slowing of activation kinetics and of inhibition of inactivation produced by Ba^2+^ on S338F mutant channels is smaller compared to WT KCNQ1. Hence, Ba^2+^ produces a twofold slowdown of activation with S338F KCNQ1 versus 19-fold with WT KCNQ1 and a 4.3-fold inhibition of inactivation with S338F KCNQ1 versus 7.8-fold with WT KCNQ1 (fig. S7, D and E, and Fig. 1, H and I). The decrease in channel inhibition fraction as a function of voltage also differed between S338F KCNQ1 and WT KCNQ1 (fig. S7F).

**Fig. 6.**
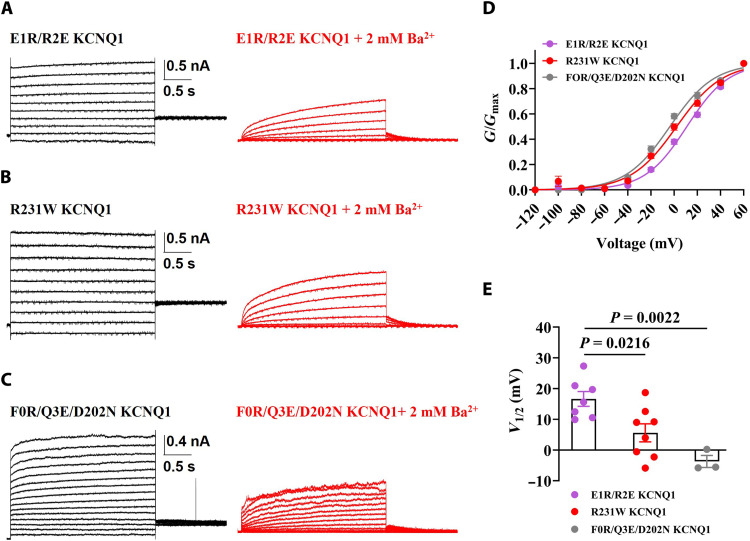
Voltage dependence of Ba^2+^ block varied in constitutively open KCNQ1 mutant channels in CHO cells. (**A** to **C**) Representative current traces for constitutively open KCNQ1 mutant channels, E1R/R2E (A), R231W (B), and FOR/Q3E/D202N (C) in the absence (black) and presence of 2 mM Ba^2+^ (red) with an external solution containing 2 mM K^+^. Starting from a −90-mV holding potential, the cell membrane stepped from −120 to +60 mV in 20-mV increments for 3 s and then repolarized for 1.5 s at −60 mV. (**D**) *G*-*V* relationships of constitutively open KCNQ1 mutant channels after 2 mM Ba^2+^. *V*_1/2_ (mV): 16.6 ± 2.2 for E1R/R2E KCNQ1 (*n* = 7), 5.6 ± 2.7 for R231W KCNQ1 (*n* = 8), and −3.7 ± 1.6 for F0R/Q3E/D202N KCNQ1 (*n* = 3). The color code is the same for (E). (**E**) *V*_1/2_ among constitutively open mutant channels after 2 mM Ba^2+^.

To more directly compare the results obtained in *Xenopus* oocytes with those gained in CHO cells, we measured, under the same experimental conditions, the effects of 2 mM external Ba^2+^ on mutant KCNQ1 channels that are constitutively open, such as the E1R/R2E, F0R/Q3E/D202N, and R231W in 2 mM external K^+^ solution. R231 is the second conserved arginine (R2) in the KCNQ1 S4 segment, and it interacts with the charge transfer center to transition the channel from the resting state to IO and AO open states (*1*, *41*, *62*, *63*). At resting potentials, R231 prevents the KCNQ1 channel from opening, and mutations at this residue produce constitutively open channels. In humans, many R231 mutations cause gain-of-channel function with clinical phenotypes such as atrial fibrillation (*64*, *65*). Similar to the results obtained in *Xenopus* oocytes, we found that exposure of all mutant channels to external Ba^2+^ produced a voltage-dependent block, thereby changing the instantaneous current activation and the linear current-voltage (*I*-*V*) relations into slowly activating, time-dependent currents and outwardly rectifying *I*-*V* relations, accompanied by the appearance of tail currents (Fig. 6, A to C, and fig. S8, A to C). We measured the *G*-*V* relationships and found that *V*_1/2_ values were differed among all the constitutively open channels (Fig. 6, D and E), again suggesting that the pore conformation in these various channels may differ and result in different binding modes of Ba^2+^ to the selectivity filter (SF) or in distinct interactions of Ba^2+^ with the permeant K^+^ in the pore.

**Fig. 7.**
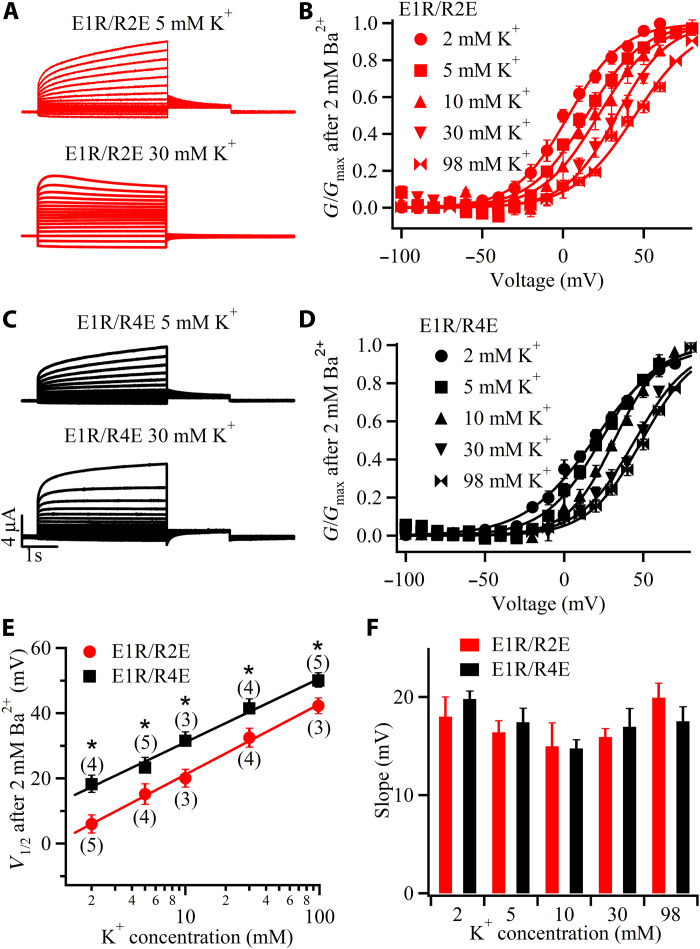
Effects of external K^+^ concentrations on voltage dependence of Ba^2+^ block in constitutively open KCNQ1 mutant channels in *Xenopus* oocytes. (**A**) Representative current traces for E1R/R2E KCNQ1 in the presence of 2 mM Ba^2+^ in bath solution containing 5 mM K^+^ (top) or 30 mM K^+^ (bottom). The channels held at −80 mV were opened from −100 to +60 mV in 10-mV increments and then subjected to a test pulse of −40 mV for tail current recording in 5 mM external K^+^ (for 30 mM external K^+^, a −80-mV test pulse was used to measure the tail current). (**B**) *G*-*V* relationships of E1R/R2E KCNQ1 in the presence of 2 mM Ba^2+^ in bath solution with varying K^+^ concentrations. (**C**) Representative current traces for E1R/R4E KCNQ1 in the presence of 2 mM Ba^2+^ in bath solution containing 5 mM K^+^ (top) or 30 mM K^+^ (bottom). Voltage protocols matched those in (A). (**D**) *G*-*V* relationships of E1R/R4E KCNQ1 in the presence of 2 mM Ba^2+^ in bath solution with varying K^+^ concentrations. (**E**) *V*_1/2_ after 2 mM Ba^2+^ in response to varying external K^+^ concentrations. Two lines were fitted with *V*_1/2_ = *A Lg* ([K^+^]) + *B*, where *A* = 21.6 and 19.6 and *B* = −0.5 and + 11.5 for E1R/R2E and E1R/R4E, respectively. *n* = 3 to 5; *P < 0.05. (**F**) Slope factor after 2 mM Ba^2+^ in response to varying external K^+^ concentrations.

The unblock of the channel at depolarized voltages is likely due to a “knock-off” of the Ba^2+^ from its binding sites by voltage and outward K^+^ permeation (*49*–*52*). To test this hypothesis, we measured voltage-dependent opening of the E1R/R2E and E1R/R4E KCNQ1 channels expressed in *Xenopus* oocytes in the presence of 2 mM external Ba^2+^ and various concentrations of external K^+^ (Fig. 7, A to D). With increasing K^+^ concentrations, less currents were blocked; particularly, the inward currents at negative voltages increased (Fig. 7, A and C). This result is consistent with the mechanism that the external K^+^ knocks off Ba^2+^ and the ions exit the channel to the cytosol (*49*, *50*, *52*). The outward current increased with depolarization voltages, and with time, and this current increase was due to the voltage- and time-dependent unblock of the fraction of the channels that were blocked by Ba^2+^ at the holding potential in each of the external K^+^ concentrations. We measured the voltage dependence of the current increase by using the time-dependent tail current to construct a *G*-*V* relationship in each K^+^ concentration (Fig. 7, B and D)

$$P_{o}=1/\left[1+\text{exp}\;\left(V-V_{1/2}\right)/s\right]$$ (1)

where *P*_o_ is the increased open probability, *V* is voltage, *V*_1/2_ is the voltage where *G*-*V* is half maximum, and *s* is the slope factor. Because the voltage- and time-dependent increase of channel opening derived from Ba^2+^ unblock, *P*_o_ is equivalent to the probability of channel unblock, *P*_unblock_, as well as the probability of Ba^2+^ unbinding of the channel, *P*_Ba unbinding_. The *G*-*V* relationship shifted to more positive voltages with increasing K^+^ (Fig. 7, B and D). The *G*-*V* shift exhibited a dependence on the logarithm of K^+^ concentration (Fig. 7E)

$$V_{1/2}=A\;\mathrm{Lg}\;\left({\left[K^{+}\right]}_{o}\right)+B$$ (2)

which is consistent with the mechanism that K^+^ knocks off Ba^2+^ block in the open channel with the force that is proportional to the electrochemical driving force for K^+^ flux through the open channel. The *V*_1/2_ dependence on K^+^ concentration exhibited a similar steepness, *A*, for E1R/R2E and E1R/R4E channels (Fig. 7E), suggesting that the mutations did not alter the nature of K^+^ flux or its interaction with Ba^2+^. The major difference of *V*_1/2_ derived from the constant *B* (Fig. 7E), which may reflect the intrinsic free energy of Ba^2+^ binding to the channel. The slope of the *G*-*V* relationships did not exhibit differences between the two channels or among various K^+^ concentrations (Fig. 7F), suggesting little variance of the membrane voltage on Ba^2+^ in the pore. These results suggest that the mutations in the VSD altered the binding of Ba^2+^ to the channel.

Because Ba^2+^ unblock depended on external K^+^ concentration, we considered the possibility of K^+^ accumulation resulting from outward currents during the measurements, particularly when external K^+^ concentration was low. Such K^+^ accumulation would contaminate the results of our measurements. Nevertheless, the *V*_1/2_ of *G*-*V* relationship exhibited similar differences between the two channels in all K^+^ concentrations (Fig. 7E), indicating no external K^+^ accumulation because the effects of K^+^ accumulation, if any, would have affected the results more at lower external K^+^ concentrations. To further rule out the possibility that the outward K^+^ current caused K^+^ accumulation that altered the *G*-*V* relationships among different channels, we measured the reversal potential of different channels in 2 mM external K^+^ in the presence of 2 mM Ba^2+^. In CHO cells, the reversal potentials of constitutively open channels, E1R/R2E, R231W, and KCNQ1 + KCNE3 in the absence and presence of 2 mM external Ba^2+^ were similar and in agreement with the expected Nernst potential values (fig. S8, D to G). In *Xenopus* oocytes, the reversal potentials of E1R/R2E, E1R/R4E, and F0R/Q3E/D202N in 2 mM external K^+^ and 2 mM Ba^2+^ were also similar (fig. S9). These results indicate that the observed differences in *G*-*V* relationships derived from the properties of the channels per se, while no evidence indicated any external K^+^ accumulation.

These results suggest that the voltage dependence of Ba^2+^ block, the binding of Ba^2+^ in the pore, and thus the conformation of the pore flexibly depend on the various interactions of the VSD with the pore. When these interactions were perturbed by the mutations in the VSD, or by the association with KCNE subunits, or when the mutations more directly changed the VSD-pore coupling, then the pore may adopt different conformations, albeit the conformational changes may be small.

## DISCUSSION

In the present study, we examined the effects of external Ba^2+^ as a K^+^ analog to probe the permeation and gating properties of KCNQ1 channels expressed in both CHO cells and *Xenopus* oocytes. The Pauling ionic radius of Ba^2+^ (1.35 Å) is very close to that of K^+^ (1.33 Å). As initially studied in the bacterial KcsA K^+^ channel, the SF structure, formed along the central axis of the homotetrameric protein, is essentially a narrow tunnel of 15 Å in length, lined by backbone carbonyl oxygen atoms pointing toward the central pore lumen that forms four K^+^ binding sites, numbered positions 1 to 4 (P1 to P4) from the extracellular to the intracellular channel side (*66*, *67*). Under physiological conducting conditions, two of these sites on average bind K^+^ ions in P1, P3 or P2, and P4 configurations. The remarkable geometrical match of these sites to the diameter of a dehydrated K^+^ ion, 2.7 Å, suggests that K^+^ selectivity emerges from “snug-fit” of the preferred ion (*68*–*71*). Ba^2+^-soaked crystal structures of WT KcsA in the absence of K^+^ show two distinct Ba^2+^ densities within the SF of the open-gate conformation: a main inner site coincident with the P4 K^+^ binding site near the internal end of the SF, and a site close to P2 (*53*, *71*–*73*). It is assumed that Ba^2+^ ions do not simultaneously occupy both sites. Once inside the SF, a single Ba^2+^ ion likely alternates back and forth between P4 and P2 sites before leaving the channel to terminate the block event (*53*). Due to its increased charge, once bound to the SF, Ba^2+^ blocks the channel conduction of K^+^ (*49*, *50*).

Based on Ba^2+^ blocking kinetics, we previously showed that, in KCNQ1, there is a “slow deep” Ba^2+^ site that occludes voltage-dependently the channel pore and a “fast superficial” Ba^2+^ site that may affect channel gating (*45*, *46*). In the present study, we show that, in K^+^ solutions, the Ba^2+^ affinities for the two sites measured at steady state likely overlap since the EC_50_ values are roughly within the same order of magnitude, although we showed that from −30 up to 0 mV, the best fit corresponds to two binding sites (Fig. 1D). The Ba^2+^ block of KCNQ1 is highly voltage dependent, with weaker inhibition at more depolarized potentials than at hyperpolarized voltages, which is likely due to electrostatic charge repulsion. This voltage dependence of Ba^2+^ block is not only observed for WT KCNQ1 but also for I_Ks_ and KCNQ1 mutants such as S338F and F351A and accounts for the significant right-shift in the voltage dependence of channel activation (Figs. 1, 2, and 5 and figs. S6 and S7). In addition, the slowdown of activation kinetics reflects the slow unblock of Ba^2+^, predominant at depolarized potentials, while the speed up of deactivation kinetics likely arises from the faster Ba^2+^ reblock than the channel closure at negative tail voltages. The voltage dependence of Ba^2+^ block is also revealed by the change of constitutively open channels such as E1R/R2E KCNQ1, F0R/Q3E/D202N KCNQ1, E1R/R4E KCNQ1, E1R/R4E KCNQ1 + KCNE1, KCNQ1 + KCNE3, and R231W KCNQ1 into time- and voltage-dependent delayed-rectifier K^+^ currents with an appearance of tail currents (Figs. 3, 4, and 6 and figs. S3 and S8, A to C). The voltage-dependent Ba^2+^ unblock kinetics could be fitted with a two-exponential function (Fig. 3H), which is consistent with the two-sites Ba^2+^ block. Our previous studies showed that, similar to other K_V_ channels (*13*, *74*), the VSD of KCNQ1 activates in resolvable steps: from the resting (R) state to an intermediate (I) state, and then to a fully activated (A) state. However, unlike Shaker-like K_V_ channels that open only when all VSD are fully activated (*13*, *74*), KCNQ1 channel is open at both the intermediate (I) and fully activated (A) VSD states, resulting, respectively, in an IO and AO open state (*37*, *39*–*41*, *54*, *55*, *75*–*77*). Previous studies demonstrated that external Ba^2+^ does not alter the gating current in the Shaker K^+^ channel, as measured by the cut-open Vaseline gap voltage clamp (*51*), nor does it affect the fluorescence change representing the VSD movement in KCNQ1-psWT (C214S/V221C/C331S) by VCF (*78*). Our results further revealed that external Ba^2+^ does not interfere with the two-step gating motion of the VSD nor disrupt its function when the VSD is locked in the intermediate state as demonstrated by the VCF experiments (Figs. 2 and 3).

While external Ba^2+^ does not directly alter VSD activation, we found that the voltage dependence of Ba^2+^ block, as measured by voltage-dependent channel opening in the presence of Ba^2+^ (Figs. 4, 6, and 7) and voltage-dependent Ba^2+^ inhibition fraction (Fig. 5), varies with the changes in VSD, VSD-pore coupling, and association of KCNE regulatory subunits. These observations and the results of voltage-dependent channel opening in the presence of Ba^2+^ in various external K^+^ (Fig. 7) indicate that Ba^2+^ interactions with the SF and the permeant ion K^+^ are sensitive to and influenced by subtle changes in VSD conformations and KCNE regulatory subunits that are located outside of the pore (fig. S2), which demonstrated that in a voltage-gated ion channel, the properties of the open pore and ion permeation may be constantly influenced by the gating state. Crystallographic data of a K^+^ channel have demonstrated that the entry of a K^+^ ion into the middle of the filter at positions 2 and 3 is associated with a specific conformational change of the SF (*71*). It was suggested that readjustment of protein atoms occurs up to a distance of 15 Å from the ion permeation pathway (*71*), indicating that the SF is not rigid and is subject to local motions of the carbonyl oxygen or small fluctuations of the backbone, both of which can be triggered by thermal fluctuations. Our data suggest that a given KCNQ1 mutant or KCNE subunit association triggers VSD activation to different states that allosterically couple to subtle changes in the conformation of the flexible SF, thereby resulting in differences in Ba^2+^ block properties. Considering that Ba^2+^ block changed when the VSD was arrested in different states along the activation pathway, the intermediate (E1R/R2E, F0R/Q3E/D202N) and the fully activated (E1R/R4E) (Fig. 4 and fig. S2), a more intriguing possibility is that the conformation of the SF may change while the VSD moves from the resting state to the fully activated state during channel activation.

Together, our results show that Ba^2+^ does not only act as a pore blocker but also functions as a remarkable sensor that discriminates the subtle changes in the conformation of the flexible SF resulting from the coupling of different VSD activation states that occur with KCNQ1 mutations or KCNE subunit association. Whether this change in the SF alter single channel conductance is not clear. Single channel recordings from KCNQ1 and I_Ks_ channels revealed the complex subconductances during the opening steps, during inactivation, and during regulation by associated KCNE1 subunit (*37*, *39*, *79*–*81*). However, a previous study showed that E1R/R2E and E1R/R4E mutant channels that respectively lock KCNQ1 in the IO and AO states had a similar single-channel conductance of ~0.18 pS (*39*). Nevertheless, we cannot exclude that changes in Ba^2+^ affinity may be imparted by coupling structural changes distal from the SF Ba^2+^ sites by modulating K^+^ flux dynamics, although this possibility is unlikely because Ba^2+^ block in different mutant KCNQ1 channels exhibited a similar dependence on K^+^ flux (Fig. 7).

Our previous studies suggested that some mutations in the VSD may alter many properties of the KCNQ1 channel. For instance, E1R/R2E and E1R/R4E channels differ in current size, ion permeability, phosphatidylinositol 4,5-bisphosphate (PIP_2_) sensitivity (*37*), sensitivity to adenosine 3′,5′-cyclic monophosphate (cAMP) modulation (*82*), and sensitivity to modulating compounds XE991 and ML277 (*37*, *54*) because these two mutations arrest the channel in the IO and AO states, respectively. On the other hand, mutations E1R/R2E and F0R/Q3E/D202N both arrest the channel in the IO state and exhibited little measurable difference (*41*). In all these channels, Ba^2+^ block and unblock exhibited different voltage dependence (Figs. 4, 6, and 7), suggesting that Ba^2+^ binding was altered by the conformational changes due to these mutations, rather than indirectly by the influence of other properties. Our measurements of voltage-dependent Ba^2+^ block and unblock were based on the time-dependent tail currents (Figs. 3, 4, 6, and 7), which only represented open channel Ba^2+^ block. Therefore, these measurements should not be affected by the opening or closing of the activation or inactivation gate that may differ in different mutant channels.

The VSD conformational changes that propagate through the trajectory VSD-S4S5 linker-S5S6 segment-SF or some other trajectories to the SF of KCNQ1 likely provide a coupling between the gating machinery and the permeation pathway, for which Ba^2+^ ions can discriminate the tiny differences in pore conformations. In KCNQ1 channels, the VSD-pore coupling follows a hand-and-elbow model, such that the coupling mechanism changes when VSD activates from the resting to the intermediate state and then to the activated state (*40*). PIP_2_ also participates in the VSD-pore couplings (*48*, *83*). Two PIP_2_ molecules bind to each KCNQ1 subunit, one at the VSD (V-PIP_2_) and the other at the interface between the VSD and the pore (C-PIP_2_) (*3*, *83*). Both sites for PIP_2_ binding change when the VSD activates from the intermediate state to the fully activated state (*83*). Thus, PIP_2_ effects on VSD-pore coupling in KCNQ1 also vary during VSD activation from the resting to intermediate and the activated states. These differences may contribute to our observed changes in Ba^2+^ binding. However, these observed differences only occur in the coupling from the VSD to the activation gate, but how the activation gate, located at the cytosolic side of the pore domain, is coupled to the SF is not clear. The couplings between the SF and other parts of the pore were observed for the K^+^ channels lacking the VSD. For instance, in tandem of P-domains in a weak inward rectifying K⁺ channel (TWIK)-related potassium channel 2 (TREK-2), a two-pore domain potassium (K_2P_) channel, movements of the proximal C terminus control the stability (i.e., conductivity) of the filter gate (*84*). TREK-2 channel was also shown to be allosterically activated at the SF by binding of the small molecule 2-aminoethoxydiphenyl borate to the proximal C terminus (*85*). More recently, it was shown that G_βγ_ activation of G protein-gated inwardly rectifying potassium 2 (GIRK2) channels is accompanied by an ~2.5-fold decrease in the affinity of Ba^2+^, compared to GIRK2 channel alone (*86*). In these channels, the coupling occurs within the structure of the pore per se, but the mechanism underlying such couplings is not clear.

While it has not been reported previously that the voltage sensor state altered the properties of an open pore of voltage-gated channels such as Ba^2+^ block, there have been ample studies to show that mutations of the SF in K_V_ channels altered voltage-dependent gating (*22*, *87*). In some K_V_ channels, C-type inactivation (*88*) involves a dilation at the extracellular part of the SF (*89*–*92*), and the development of which is accompanied with voltage sensor movements (*93*–*95*). In voltage-gated Na_V_ channels, the slow inactivation, which mechanistically resembles that of the C-type inactivation, was also found to correlate with domain IV VSD activation (*96*). These types of inactivation are physiologically important and may develop slowly, long after the voltage sensors have been fully activated and the activation gate opened at a constant depolarization (*89*). However, the onset of the SF dilation in these types of inactivation is still not known. A recent study suggested that the SF dilation may occur spontaneously after pore opening, which may be set up by structural rearrangements in and around the SF during and after activation (*92*). It was shown that in Shaker H4 inactivation removed (IR) channels, Ba^2+^ block affects the C-type inactivated state by speeding the off-gating current in W434F mutant channels, while the mutations of D447 and T449, which are critical for C-type inactivation (*89*, *92*), altered Ba^2+^ block (*97*). These results indicate that Ba^2+^ interaction with the SF is sensitive to the changes in Shaker C-type inactivation. If it is common in voltage-gated channels that voltage sensor movements during activation alter SF, then it is possible that this mechanism may contribute to initiating the process of these types of inactivation.

## MATERIALS AND METHODS

### Constructs and site-directed mutagenesis

Human WT *KCNQ1*, *KCNE1*, and *KCNE3* cDNA were cloned separately into the pcDNA 3.1 (+) vector (catalog no. V79020, Thermo Fisher Scientific). Plasmid DNA was amplified in *Escherichia coli* DH5α competent cells. Overlap extension and high-fidelity polymerase chain reaction were used to generate mutations in the WT *KCNQ1* cDNA in the pcDNA 3.1 (+) vector (catalog no. V79020, Thermo Fisher Scientific). WT *KCNQ1*, WT *KCNE1*, WT *KCNE3*, and all *KCNQ1* mutants were verified through Sanger DNA sequencing in both directions. The quality of the plasmid DNA was assessed by agarose gel electrophoresis and Nanodrop. The diluted plasmid DNA was stored at −20°C until further use.

### Cell culture and transfection for channel expression

CHO (catalog no. CCL-61, American Type Culture Collection) cells were grown in Dulbecco’s modified Eagle’s medium (catalog no. D5671, Sigma-Aldrich) supplemented with 2 mM l-glutamine (catalog no. G7513, Sigma-Aldrich), 10% (v/v) fetal bovine serum (catalog no. F9665, Sigma-Aldrich), and 1% (v/v) penicillin-streptomycin (catalog no. 03-031-5C, Biological Industries). In brief, CHO cells were plated at a density of 40,000 cells per well on poly-l-lysine (10 μg/ml, catalog no. P2636, Sigma-Aldrich) treated glass coverslips (13 mm in diameter, catalog no. BNCB00130RA1N, Bar Naor) in a 24-well sterile tissue culture plate. CHO cells were transfected with a mixture of 0.5 μg of WT or mutant *KCNQ1*, 0.18 μg of a plasmid encoding enhanced green fluorescent protein (EGFP) as a fluorescent marker, and *Trans*IT-LT1 Transfection Reagent (catalog no. MIR 2304, Mirus Bio) according to the manufacturer’s protocol.

### Whole-cell patch-clamp recording from CHO cells

Recordings were performed using the whole-cell configuration of the patch-clamp technique on those EGFP-positive CHO cells at room temperature (22° to 24°C). Signals were amplified using a MultiClamp 700B amplifier (Axon Instruments), sampled at 5 kHz, and filtered at 2.4 kHz via a low-pass four-pole Bessel filter. Data were acquired using pClamp 10.5 software (Axon Instruments) in conjunction with a Digidata 1440A Low-Noise Data Acquisition System (Axon Instruments). Glass pipettes were pulled from borosilicate glass (with filament, catalog no. 30-0044, Harvard Apparatus), with a pipette resistance of 3 to 5 MΩ in the presence of pipette and bath solutions. The pipette solution contained (in millimolar): 130 KCl, 5 K_2_-ATP, 5 K_4_BAPTA (no added CaCl_2,_ nominally 0 free Ca^2+^ concentration), and 10 Hepes (pH 7.3 adjusted with KOH; 294 mosmol). The bath solution contained (in millimolar): 140 NaCl, 4 KCl, 1.8 CaCl_2_, 1.2 MgCl_2_, 11 glucose, and 5.5 Hepes (pH 7.3 adjusted with NaOH; 310 mosmol). Pipette offsets were always corrected. Series resistances ranging from 8 to 12 MΩ were electronically compensated by 85% for voltage-clamp recording. Starting from a holding potential of −90 mV, cells were subjected to steps of test pulse voltage for 3 s, increasing by 10-mV increments. After each test pulse, the voltage was returned to −60 mV for 1.5 s to record the tail current. The specific test pulse voltage range for each channel is indicated in the steady-state *I*-*V* relationships. Bath solution containing a given concentration of BaCl_2_ was perfused to the recording chamber using a MINIPULS 3 Peristaltic Pump (Gilson Incorporated) with flow rate around 3.5 ml/min. The steady-state pore block was reached within 3 min after Ba^2+^. All chemicals were obtained from Sigma-Aldrich (St. Louis, MO).

### Electrophysiology data analysis for whole-cell patch-clamp recordings

Electrophysiological data analysis was performed using Clampfit (Axon Instruments), Excel (Microsoft), and GraphPad Prism (GraphPad Software) software. Leak subtraction was performed off-line with Clampfit software.

To plot the steady-state *I*-*V* relationship before and after Ba^2+^, steady-state current amplitudes at the end of each test pulse were normalized to the corresponding cell capacitance, and current densities are reported (p*A*/p*F*) as a function of voltage. The voltage-dependent block or inhibition fraction by Ba^2+^, calculated as (*I* − *I*Ba^2+^)/*I*, was determined by subtracting the steady-state current density measured after 2 mM Ba^2+^ (*I*Ba^2+^) from the original steady-state current density (*I*) and then divided by the original steady-state current density (*I*). The voltage-dependent Ba^2+^ block or inhibition fraction-voltage relationship was created by plotting the inhibition fraction against its corresponding voltage. To calculate the concentration and voltage dependence of Ba^2+^ blockage on WT KCNQ1, the one-site binding curves were best fitted using the following equation: *Y* = Bottom + (Top − Bottom)/{1 + 10^[(logIC_50_ − *X*) × HillSlope]}, where *X* values are logarithms of concentrations and Top and Bottom are plateaus in the units of the *Y* axis. For two-site binding, curves were fitted with the following equations: logEC_50_Lo = log[10^logKiLo × (1 + HotNM/HotKdNMLo)] for the low-affinity site and logEC_50_Hi = log[10^logKiHi × (1 + HotNM/HotKdNMHi)] for the high-affinity site. The IC_50_ values are reported at various voltages. Chord conductance (*G*) was calculated by using the following equation: *G* = *I*/(*V* − *V*_rev_), where *I* corresponds to the steady-state current amplitude measured at the end of each test pulse, *V* is the test pulse voltage, and *V*_rev_ is the calculated reversal potential assumed to be −90 mV in CHO cells. Normalized conductances were plotted as a function of voltage and fitted with a single Boltzmann equation in the form of *G*(*V*) = 1/{1 + exp [−(*V* − *V*_1/2_)/*k*]}, where *V* is the test pulse voltage, *V*_1/2_ indicates the half-activation voltage, and *k* is the slope factor. To calculate the concentration dependence of Ba^2+^ effect on Δ*V*_1/2_ (*V*_1/2_ after Ba^2+^ − *V*_1/2_ before Ba^2+^) and channel activation (or pore opening) kinetics (expressed as *T*_50_, which refers to the duration required for WT or mutant KCNQ1 to achieve 50% of its maximum amplitude), curves were best fitted using the following equation: *Y* = Bottom + (Top − Bottom)/{1 + 10^[(logEC_50_ − *X*) × HillSlope]}, where *X* values are logarithms of concentrations and Top and Bottom are plateaus in the units of the *Y* axis. The EC_50_ values are reported. Inactivation was quantified by the ratio of the lowest current amplitude after the decay of the transient component (dip) to the steady-state current amplitude at +60 mV, and the result was expressed as a percentage. To calculate the concentration dependence of Ba^2+^ effect on channel inactivation measured at +60 mV, curve was best fitted using the formula that calculates the concentration and voltage dependence of the Ba^2+^ effect on WT KCNQ1 block. The IC_50_ value is reported.

### Channel expression in *Xenopus* oocytes

*Xenopus* ovarian lobes containing oocytes at stage V or VI were obtained from WT *Xenopus laevis* purchased from Xenopus 1 by laparotomy, following the protocol approved by the Washington University Animal Studies Committee (protocol no. 20190030). The collected ovarian lobes were divided into clumps, each containing around 10 oocytes. Then, they were digested by type IA collagenase from *Clostridium histolyticum* (catalog no. C9891, lot no. 0000142669, Sigma-Aldrich) to achieve the single fully defolliculated oocytes. Only stage V or VI oocytes without a follicle cell layer were chosen for capped RNA (cRNA) microinjection. cRNA was in vitro transcribed from the linearized cDNA using the mMESSAGE mMACHINE T7 Transcription Kit (catalog no. AM1344, Thermo Fisher Scientific). The quality of the cRNA was assessed by agarose gel electrophoresis and Nanodrop. The stock and diluted cRNA were stored at −80°C until further use.

For two-electrode voltage-clamp (TEVC) recording, a total of 18.4 ng of WT or mutant *KCNQ1* cRNAs was microinjected into each oocyte through the equatorial band between the animal and vegetal poles using a Nanoject II auto-nanoliter injector (catalog no. 3-000-204, Drummond Scientific). For experiments involving coexpression with WT KCNE1 or WT KCNE3, WT *KCNE1* cRNA was coinjected with WT or mutant *KCNQ1* cRNAs at a mass ratio of 1:4; WT *KCNE3* cRNA was coinjected with WT *KCNQ1* cRNA at a mass ratio of 1:4. For VCF recording, KCNQ1-psWT, which contains three mutations (C214A, G219C, and C331A), was used to enable fluorescence labeling specifically for G219C to trace the VSD movement. A total of 18.4 ng of *KCNQ1-psWT* cRNA was microinjected. The cRNA-injected oocytes were then transferred to 48-well sterile tissue culture plates filled with 1xND96 culture solution (in millimolar): 96 NaCl, 2 KCl, 1.8 CaCl_2_, 1 MgCl_2_, 5 Hepes, 2.5 CH_3_COCO_2_Na (sodium pyruvate), and 1:100 penicillin-streptomycin (pH 7.6 adjusted with NaOH) and incubated at 18°C for at least 2 days before recording.

### TEVC and VCF recordings from *Xenopus* oocytes

Pipettes were fabricated using thin-walled borosilicate glass (with filament, catalog no. BF150-117-10, Sutter Instrument) with a micropipette puller (catalog no. P-1000, Sutter Instrument). Pipette resistance was around 0.8 MΩ when filled with a 3 M KCl solution and immersed in a 1xND96 bath solution (in millimolar): 96 NaCl, 2 KCl, 1.8 CaCl_2_, 1 MgCl_2_, and 5 Hepes (pH 7.6 adjusted with NaOH).

For TEVC experiments, whole-oocyte currents, sampled at 1 kHz and low-pass filtered at 2 kHz, were recorded with a GeneClamp 500B amplifier equipped with a virtual-ground bath clamp (Axon Instruments) driven by Patchmaster (version: v2x53, HEKA Elektronik) software. The raw current traces in fig. S9A of E1R/R4E KCNQ1 with 2 mM Ba^2+^ were again low-pass filtered at 50 Hz for illustration due to the high noise-to-signal ratio with small currents. For VCF experiments, oocytes expressing KCNQ1-psWT channels were labeled for 30 min on ice with 10 μM Alexa Fluor 488 C5 maleimide (catalog no. A10254, lot no. 2885718, Thermo Fisher Scientific) in high K^+^ solution (in millimolar): 98 KCl, 1.8 CaCl_2_, and 5 Hepes (pH 7.6 adjusted with NaOH). The oocytes were then washed three times with 1xND96 bath solution and kept on ice until recording. To simultaneously measure the current from pore opening and the fluorescence signal from VSD movement, a patch-clamp amplifier (EPC 10, HEKA Elektronik) with its headstage connected to a photodiode (catalog no. PIN-020A, OSI Optoelectronics) was linked to a GeneClamp 500B amplifier equipped with a virtual-ground bath clamp (Axon Instruments). Whole-oocyte currents from the VCF experiment were recorded in 1xND96 bath solution using the same instrument as the TEVC setup. The fluorescence emission due to VSD movement was simultaneously collected by photodiode. The amplified signals were low-pass filtered at 200 Hz and sampled at 1 kHz using Patchmaster (version: v2x53, HEKA Elektronik). Starting from a holding potential of −80 mV for 0.5 s, oocytes were subjected to steps of test pulse voltage for 4 s, increasing by 10-mV increments until the tail current saturated. After each test pulse, the voltage was returned to −40 mV for 2 s to record the tail current. The specific test pulse voltage range for each channel is indicated in the steady-state *I*-*V* relationships. All recordings were performed at room temperature (22° to 24°C), and all chemicals were obtained from Sigma-Aldrich (St. Louis, MO).

### Electrophysiology data analysis for TEVC and VCF recordings

Electrophysiology data were analyzed using MATLAB (MathWorks), Clampfit (Axon Instruments), Excel (Microsoft), OriginPro (OriginLab), and GraphPad Prism (GraphPad Software) software. The steady-state *I*-*V* relationship before and after 2 mM Ba^2+^ was created by plotting the normalized steady-state current at the end of each test pulse against its corresponding voltage. The voltage-dependent block or inhibition fraction by Ba^2+^, calculated as (*I* − *I*Ba^2+^)/*I*, was determined by subtracting the steady-state current measured in the presence of 2 mM Ba^2+^ (*I*Ba^2+^) from the original steady-state current (*I*) and then divided by the original steady-state current (*I*). The inhibition fraction-voltage relationship was created by plotting the inhibition fraction against its corresponding voltage. The voltage dependence of the channel activation or pore opening before and after 2 mM Ba^2+^ was estimated by calculating the normalized *G*-*V* relationship, which was generated by plotting the normalized instantaneous tail currents recorded at −40 mV following test pulses against the test pulses. The *G*-*V* relationship was fitted with a single Boltzmann equation in the form of *G*(*V*) = 1/{1 + exp [−(*V* − *V*_1/2_)/*k*]}, where *V* is the test pulse voltage, *V*_1/2_ indicates the half-activation voltage, and *k* is the slope factor. For VSD movement trajectory data analysis, the voltage-dependent activation of the VSD was estimated by calculating the *F*-*V* relationship. First, linear photobleaching correction was applied to each fluorescence trace by subtracting the line obtained by linear fitting of the 2-s fluorescence signal before the start of the test pulse. Next, the *F*-*V* relationship was generated by plotting the normalized change of steady-state fluorescence emission (Δ*F*/*F*_max_) at the end of the test pulses against test pulses. The *F*-*V* relationship was fitted with a double Boltzmann distribution in the form of *F*(*V*) = *A*1/{1 + exp [−(*V* − *V*_1/2,1_)/*k*_1_]} + *A*2/{1 + exp [−(*V* − *V*_1/2,2_)/*k*_2_]}, where *V* represents the test pulse voltage and *A*1 + *A*2 = 1. *V*_1/2,1_ and *V*_1/2,2_ indicate the half-activation voltages for *F1* and *F2*, respectively. *k*_1_ and *k*_2_ is the slope factor for *F1* and *F2*, respectively. To analyze the kinetics of the voltage-dependent of the channel activation or pore opening before and after 2 mM Ba^2+^, the 4-s current traces within test pulse voltages were selected. The time constants τ-fast and τ-slow were calculated by fitting the current trace within test pulse voltage to a biexponential equation in the form of *I*(*t*) = A-fast [1 − exp (−*t*/τ-fast)] + A-slow [1 − exp (−*t*/τ-slow)] + C-offset. Each current trace was baseline-corrected using the mean current at the holding potential for each trace. Time constant τ-fast- and τ-slow–voltage relationships were plotted separately. To analyze the kinetics of the tail current before and after 2 mM Ba^2+^, 2-s tail current traces at −40 mV, which originated from the +60 mV test pulse, were selected. The time constant τ was calculated by fitting the 2-s tail current to a single exponential equation in the form of *I*(*t*) = A [1 − exp (−*t*/τ)] + C-offset. Leak currents were not baseline-subtracted when calculating the *G*-*V* and *I*-*V* relationships.

### Statistical analysis

All average data are presented as means ± SEM. Some error bars representing SEM are too small and covered by the symbols. Each experiment was independently replicated at least three times, and *n* introduced in the figure legend represents the number of CHO cells (or *Xenopus* oocytes) that were analyzed. Statistical analyses were performed using GraphPad Prism (GraphPad Software). For paired analyses, a two-tailed paired *t* test was used when the differences between paired observations followed a normal distribution. Otherwise, the Wilcoxon matched-pairs signed-rank test was used. For unpaired analyses between two groups, a two-tailed unpaired *t* test was used when both groups followed a normal distribution and variances were equal. Welch’s *t* test was used when the data were normally distributed but variances were unequal. The Mann-Whitney test was performed when data were not normally distributed. For clarity and space efficiency, paired and unpaired comparisons were presented in the same panel of Fig. 3I, with statistical tests selected according to the criteria listed above. For unpaired analyses among groups, ordinary one-way analysis of variance (ANOVA) followed by Tukey’s multiple comparisons test was used when the data were normally distributed and variances were equal. Statistical results are reported in the figure legend or within the figure. A *P* value of less than 0.05 was considered statistically significant.
