# Supplementary material for: Barium senses subtle pore changes in a voltage-gated K+ channel associated with voltage sensor states and regulatory subunits
Source: Sci Adv. 2026 Jul 10;12(28):eaec6510. doi: 10.1126/sciadv.aec6510 (PMC13353409; doi:10.1126/sciadv.aec6510)
Supplement: Supplementary file 1 — Figs. S1 to S9 [file sciadv.aec6510_sm.pdf]

Science Advances
AAAS

Supplementary Materials for

Barium senses subtle pore changes in a voltage-gated K^+ channel associated with voltage sensor states and regulatory subunits

Lei Huang *et al.*

Corresponding author: Bernard Attali, battali@tauex.tau.ac.il; Jianmin Cui, jcui@wustl.edu

Sci. Adv. **12**, eaec6510 (2026)
DOI: 10.1126/sciadv.aec6510

This PDF file includes:

Figs. S1 to S9

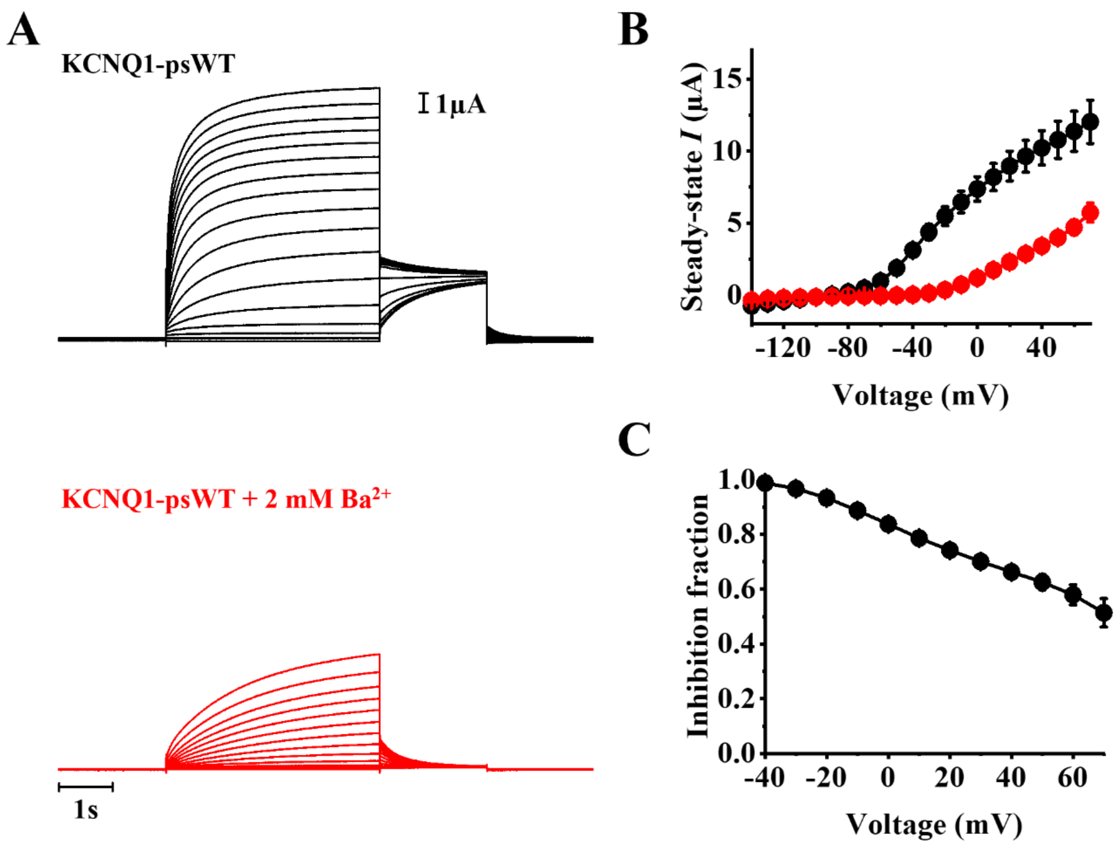

fig. S1. The effect of 2 mM external Ba^{2+} on pore opening of KCNQ1-psWT in *Xenopus* oocytes.

(A) Representative current traces from KCNQ1-psWT (C214A/G219C/C331A) expressed in *Xenopus* oocytes with recordings made in the absence (black) and presence of 2 mM Ba^{2+} (red) using VCF from the same oocyte. **(B)** I-V relationships of KCNQ1-psWT before and after 2 mM Ba^{2+} (n = 4). **(C)** Inhibition fraction of KCNQ1-psWT (n = 4).

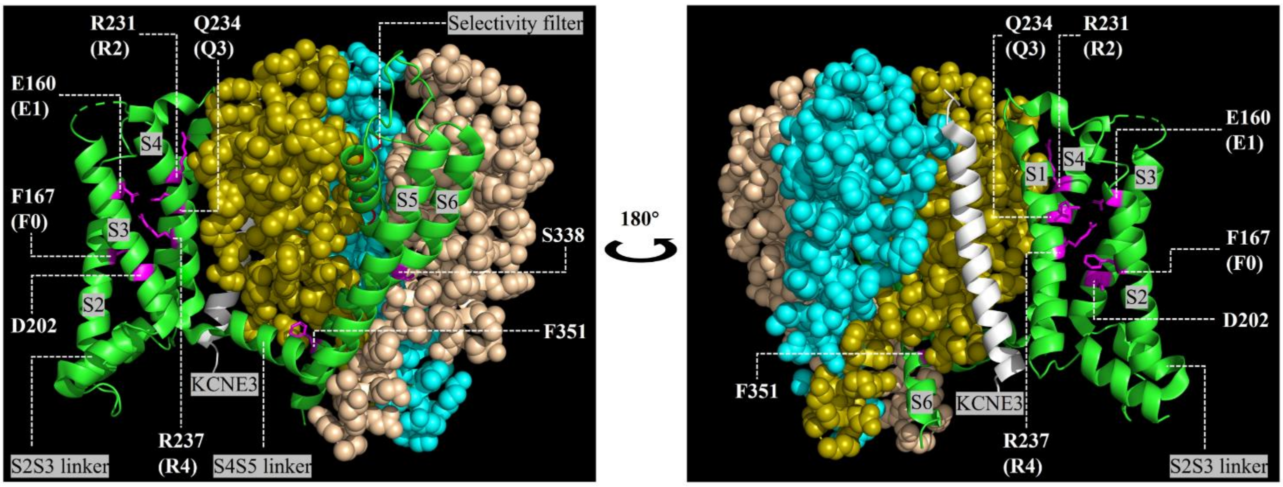

fig. S2. Mutations in KCNQ1+KCNE3.

The structure of KCNQ1+KCNE3 (Protein Data Bank Entry ID: 6V00) is shown with the pore domain of 3 KCNQ1 subunits (space filling), the VSD and the pore domain of the fourth subunit (ribbon, green, red, and magenta), and one KCNE3 subunit (white). Red represents the selectivity filter of the KCNQ1 subunit (TIGYG). Residues, including E160 (E1), F167 (F0), D202, R231 (R2), Q234 (Q3), R237 (R4), S338, and F351, are highlighted in magenta. Residues E1 and F0 are situated in the S2 segment, D202 is positioned in the S3 segment, R2, Q3, and R4 are located in the S4 segment, and S338 and F351 are located in the S6 segment. The structure below the S6 segment has been omitted. When assembled with KCNQ1, the spatial location of KCNE1 is very similar to that of KCNE3, as shown in our most recent study (*3*).

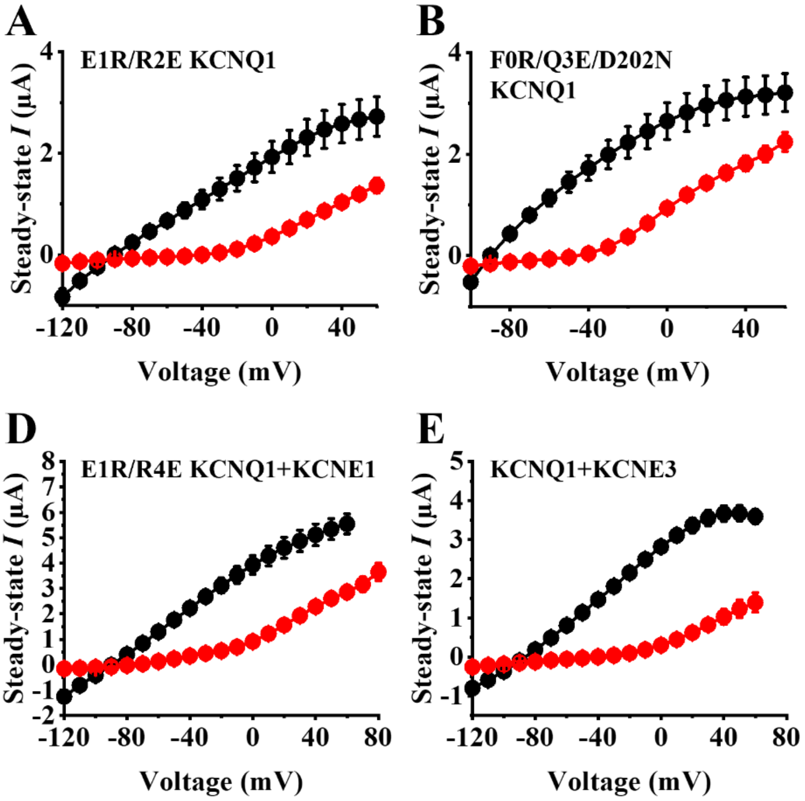

fig. S3. External Ba^{2+} reduced current amplitude of constitutively open KCNQ1 channels in *Xenopus* oocytes.

I-V relationships of constitutively open KCNQ1 channels, including E1R/R2E KCNQ1 ($n = 8$) **(A)**, F0R/Q3E/D202N KCNQ1 ($n = 5$) **(B)**, E1R/R4E KCNQ1 ($n = 8$) **(C)**, E1R/R4E KCNQ1+KCNE1 ($n = 6$) **(D)**, and KCNQ1+KCNE3 ($n = 5$) **(E)** in the absence (black) and presence of 2 mM Ba^{2+} (red).

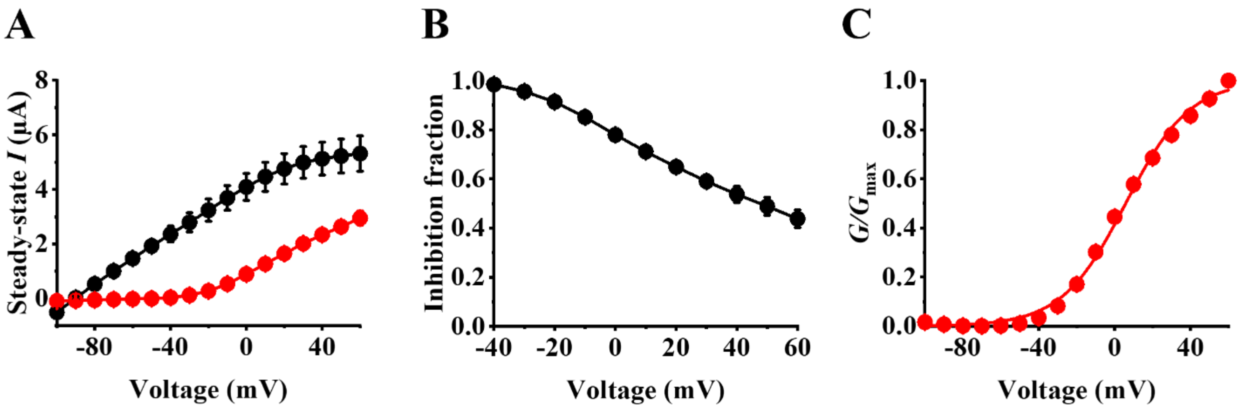

fig. S4. The effect of 2 mM external Ba^{2+} on pore opening of E1R/R2E KCNQ1-psWT in *Xenopus* oocytes.

(A) I-V relationships of E1R/R2E KCNQ1-psWT (E160R/C214A/G219C/R231E/C331A) in the absence (black) and presence of 2 mM Ba^{2+} (red) ($n = 3$). **(B)** Inhibition fraction of E1R/R2E KCNQ1-psWT ($n = 4$). **(C)** G-V relationship of E1R/R2E KCNQ1-psWT in the presence of 2 mM Ba^{2+} calculated from **Fig. 3D (red)**, which was well fitted by single Boltzmann equation with $V_{1/2}$ and Slope factor (mV) of 6.068 ± 0.935 and 16.793 ± 0.201 ($n = 4$).

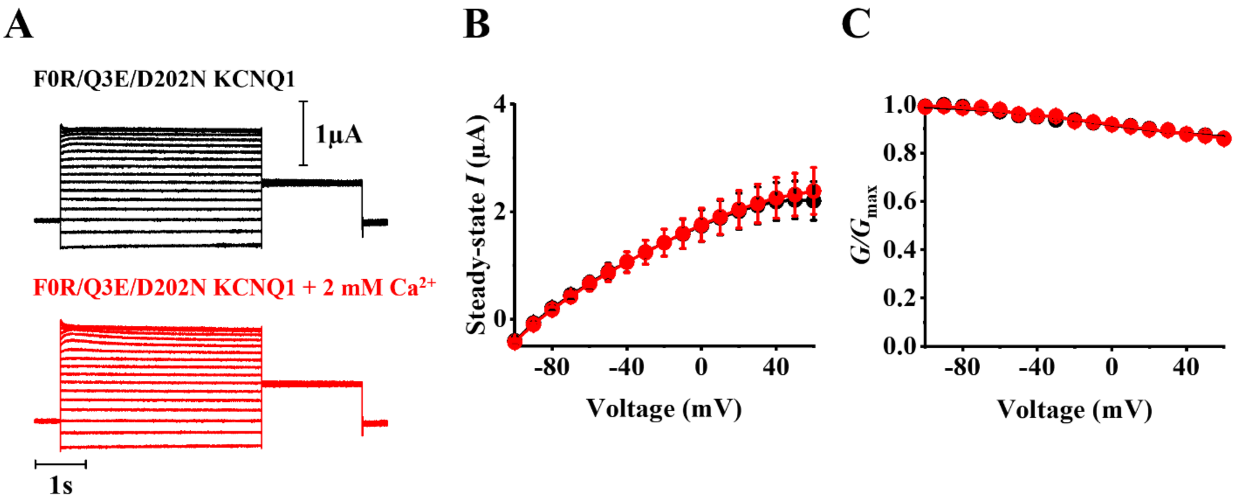

fig. S5. Not every divalent metal cation (Ca^{2+}, for example) caused a voltage-dependent block of the constitutively open KCNQ1 channel (F0R/Q3E/D202N KCNQ1, for example) in *Xenopus* oocytes.

(A) Representative current traces from the constitutively open channel F0R/Q3E/D202N KCNQ1 expressed in *Xenopus* oocytes, with recordings made in the absence (black) and presence of 2 mM external Ca^{2+} (red) from the same oocyte. **(B and C)** **(B)** I-V ($n = 4$) and **(C)** G-V ($n = 4$) relationships of F0R/Q3E/D202N KCNQ1 in the absence and presence of 2 mM Ca^{2+}.

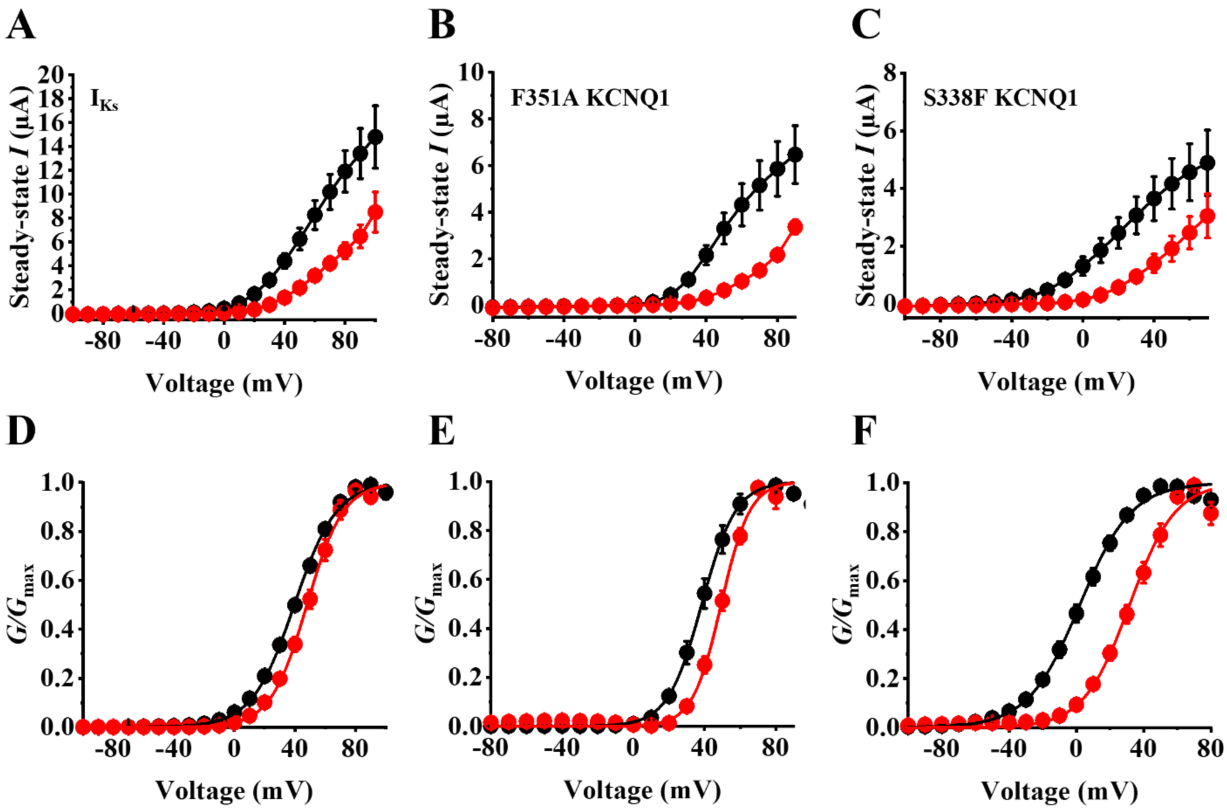

fig. S6. External Ba^{2+} reduced current amplitude and caused a rightward shift in the G-V for I_{Ks}, F351A KCNQ1, and S338F KCNQ1 in *Xenopus* oocytes.

(A-C) I-V relationships of I_{Ks} (n = 6) **(A)**, F351A KCNQ1 (n = 4) **(B)**, and S338F KCNQ1 (n = 7) **(C)** in the absence (black) and presence of 2 mM Ba^{2+} (red). **(D-F)** G-V relationships of I_{Ks} **(D)**, F351A KCNQ1 **(E)**, and S338F KCNQ1 **(F)** before and after 2 mM Ba^{2+}. $V_{1/2}$ and Slope factor (mV): 39.675 ± 1.291 and 13.809 ± 0.357 for I_{Ks} and 48.256 ± 2.065 and 12.379 ± 0.262 for I_{Ks} after 2 mM Ba^{2+} (n = 6, P = 0.0039 for $V_{1/2}$ and P = 0.0312 for Slope factor between two groups), 38.653 ± 2.527 and 9.202 ± 0.376 for F351A KCNQ1 and 49.485 ± 1.372 and 8.327 ± 0.143 for F351A KCNQ1 after 2 mM Ba^{2+} (n = 4, P = 0.0041 for $V_{1/2}$ between two groups), 2.156 ± 2.410 and 15.030 ± 0.211 for S338F KCNQ1 and 31.851 ± 2.268 and 13.713 ± 0.283 for S338F KCNQ1 after 2 mM Ba^{2+} (n = 7, P < 0.0001 for $V_{1/2}$ and P = 0.0156 for Slope factor between two groups).

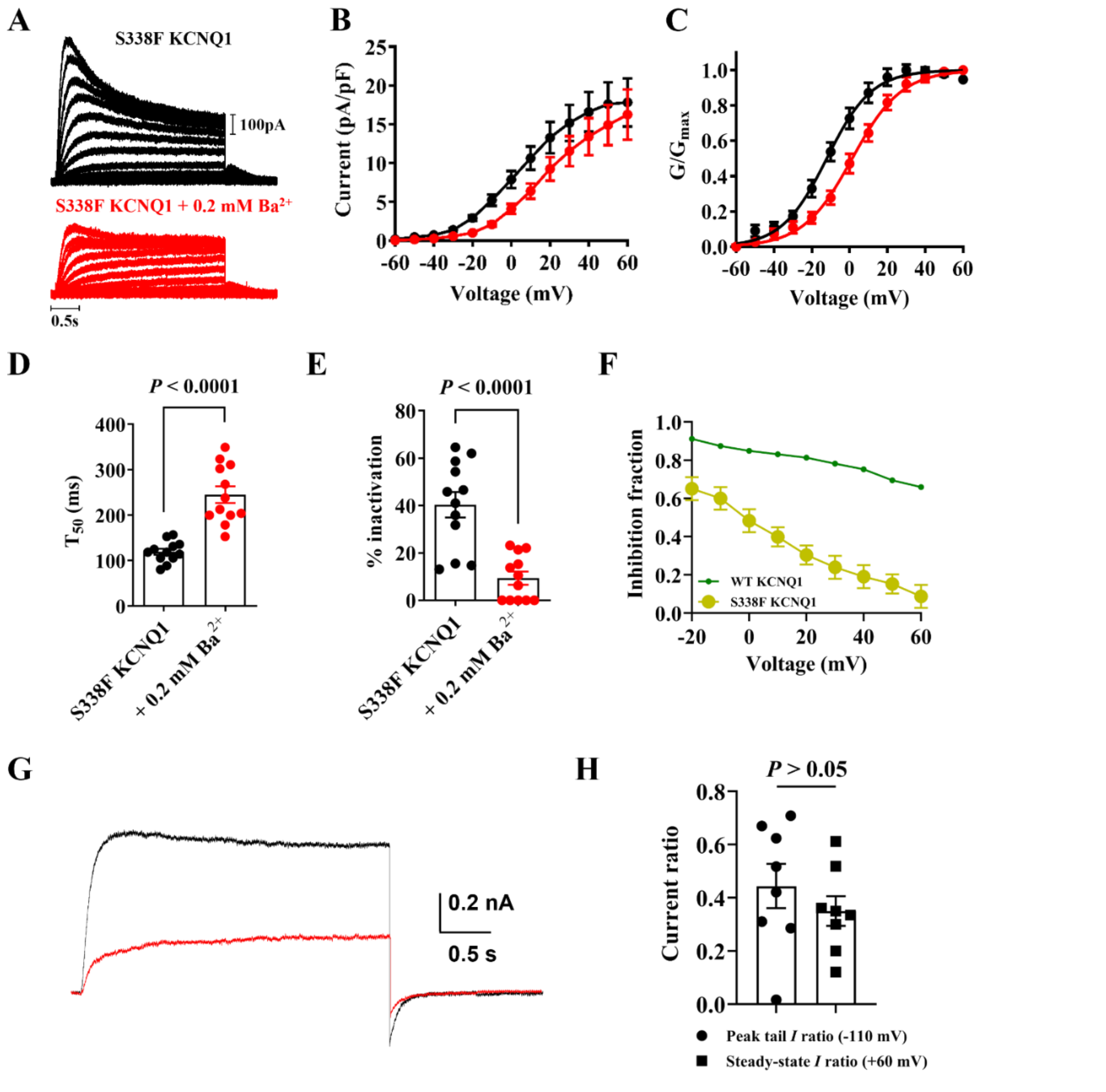

fig. S7. External Ba^{2+} causes a voltage-dependent block of S338F KCNQ1 in CHO cells.

(A) Representative S338F KCNQ1 current traces in the absence (black) and presence of 0.2 mM Ba^{2+} (red). **(B)** I-V relationships of S338F KCNQ1 before and after 0.2 mM Ba^{2+} (n = 9). **(C)** G-V relationships of S338F KCNQ1 before and after 0.2 mM Ba^{2+}. $V_{1/2}$ and Slope factor (mV): -12.1 ± 2.9 and 11.9 ± 0.9 for S338F KCNQ1, 1.4 ± 3.1 and 13.1 ± 0.6 for S338F KCNQ1 with 0.2 mM Ba^{2+} (n = 12; $P < 0.0001$ for $V_{1/2}$). **(D)** External Ba^{2+} slowed the channel opening kinetics of S338F KCNQ1 (n = 12). **(E)** External Ba^{2+} reduced the percentage of inactivation (n = 12). **(F)** Inhibition fraction of S338F KCNQ1 (n = 12). Inhibition fraction of WT KCNQ1 was reproduced from Fig. 1C. **(G)** Representative current traces obtained from the same cell in the absence (black) and presence of 2 mM Ba^{2+} (red). Starting from a -90 mV holding potential, the membrane was stepped to +60 mV for 3 seconds and then repolarized to a tail potential of -110 mV. **(H)** Current ratios measured at -110 mV peak tail (black solid circle) were similar to those calculated from +60 mV steady-state (black solid square) (n = 8; $P > 0.05$). The ratio of peak tail current with Ba^{2+} over the original peak tail current at -110 mV (black solid circle) and the ratio of steady-state current with Ba^{2+} over the original steady-state current at +60 mV (black solid square) were calculated from **(G)**.

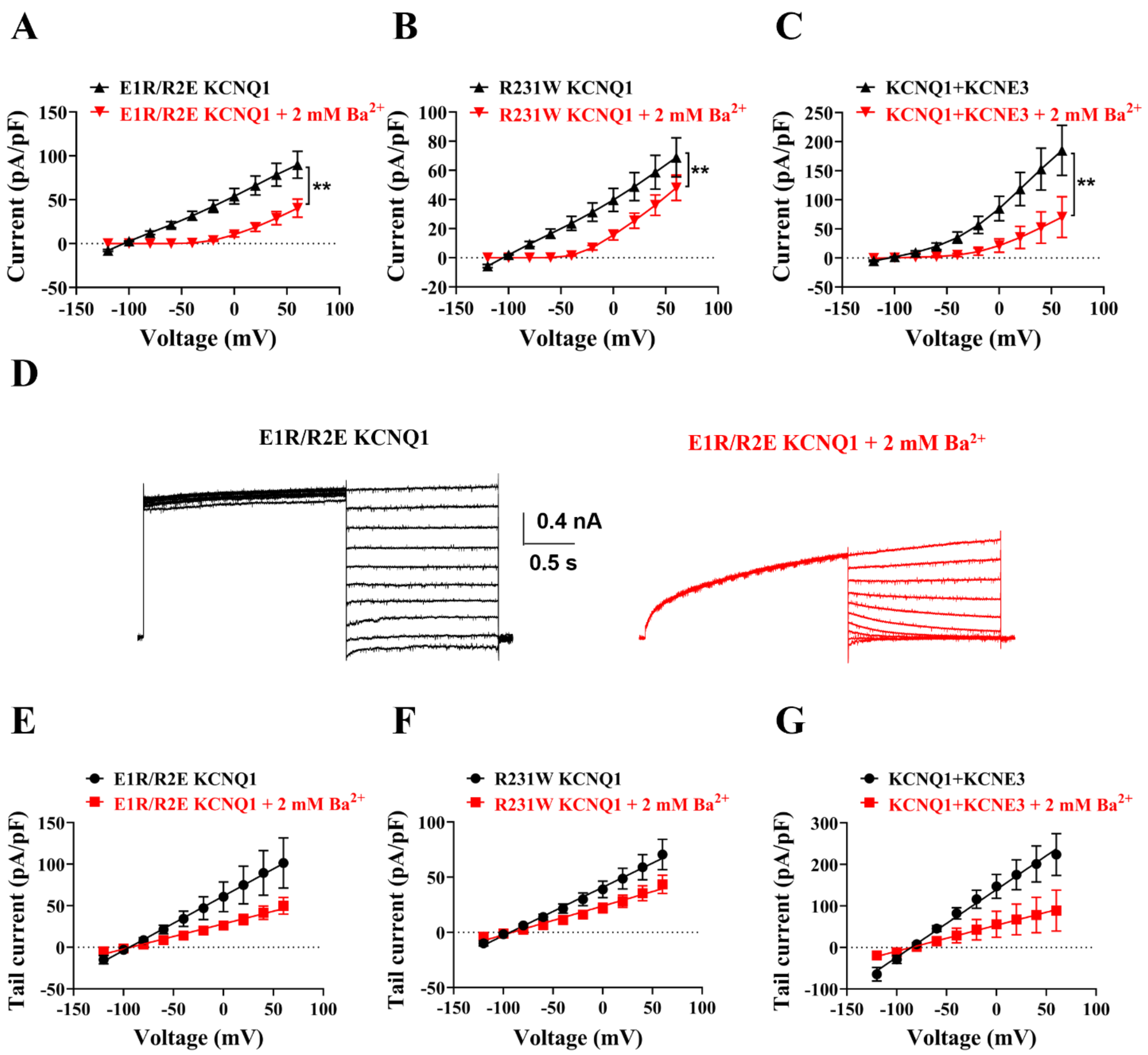

fig. S8. External Ba^{2+} blocks various constitutively open KCNQ1 channels but does not affect their reversal potential in CHO cells.

(A-C) I-V relationships of constitutively open KCNQ1 channels, including E1R/R2E KCNQ1 (n = 7; two asterisk: P = 0.0026) **(A)**, R231W KCNQ1 (n = 8; two asterisk: P = 0.0026) **(B)**, and KCNQ1+KCNE3 (n = 6; two asterisk: P = 0.0096) **(C)** in the absence (black) and presence of 2 mM Ba^{2+} (red) in CHO cells. **(D)** Representative current traces from E1R/R2E KCNQ1 constitutively open mutant channel expressed in CHO cells, recorded in the absence (black) and presence of 2 mM Ba^{2+} (red). The channels held at -90 mV were stepped to +60 mV for 2 seconds and repolarized at tail potentials from +60 to -120 mV in 20 mV decrements for 1.5 seconds. **(E-G)** Reversal potentials are consistent across all three constitutively open KCNQ1 channels in CHO cells. **(E)** Tail current-voltage relationships of E1R/R2E KCNQ1 (n = 5) showing similar reversal potential before (-94.2 mV) and after 2 mM Ba^{2+} (-93.0 mV). **(F)** Tail current-voltage relationships of R231W KCNQ1 (n = 8) showing similar reversal potential before (-93.4 mV) and after 2 mM Ba^{2+} (-91.4 mV). **(G)** Tail current-voltage relationships of KCNQ1+KCNE3 (n = 4) showing similar reversal potential before (-85.5 mV) and after 2 mM Ba^{2+} (-85.6 mV).

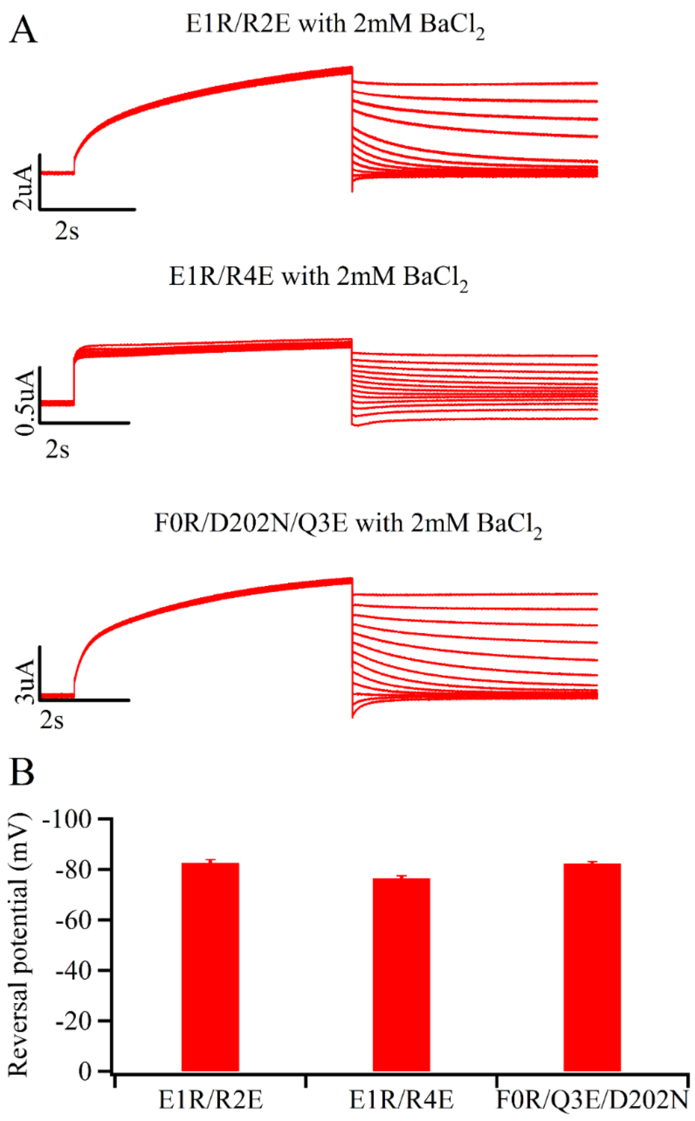

fig. S9. Reversal potential measurements for E1R/R2E, E1R/R4E, and F0R/Q3E/D202N KCNQ1 constitutively open mutant channels in *Xenopus* oocytes.

(A) Representative current traces from E1R/R2E, E1R/R4E, and F0R/Q3E/D202N KCNQ1 constitutively open mutant channels expressed in *Xenopus* oocytes, recorded in the presence of 2 mM Ba^{2+} in 1xND96 solution containing 2 mM K^{+}. The channels held at -80 mV were opened to +40 mV, and then tail currents were measured from −100 to +20 mV in 10 mV increments. **(B)** Reversal potentials are consistent across all three KCNQ1 constitutively open mutant channels ($n \geq 3$ oocytes per channel).
